# Lung cancer and socioeconomic status in a pooled analysis of case-control studies

**DOI:** 10.1371/journal.pone.0192999

**Published:** 2018-02-20

**Authors:** Jan Hovanec, Jack Siemiatycki, David I. Conway, Ann Olsson, Isabelle Stücker, Florence Guida, Karl-Heinz Jöckel, Hermann Pohlabeln, Wolfgang Ahrens, Irene Brüske, Heinz-Erich Wichmann, Per Gustavsson, Dario Consonni, Franco Merletti, Lorenzo Richiardi, Lorenzo Simonato, Cristina Fortes, Marie-Elise Parent, John McLaughlin, Paul Demers, Maria Teresa Landi, Neil Caporaso, Adonina Tardón, David Zaridze, Neonila Szeszenia-Dabrowska, Peter Rudnai, Jolanta Lissowska, Eleonora Fabianova, John Field, Rodica Stanescu Dumitru, Vladimir Bencko, Lenka Foretova, Vladimir Janout, Hans Kromhout, Roel Vermeulen, Paolo Boffetta, Kurt Straif, Joachim Schüz, Benjamin Kendzia, Beate Pesch, Thomas Brüning, Thomas Behrens

**Affiliations:** 1 Institute for Prevention and Occupational Medicine of the German Social Accident Insurance (IPA), Institute of the Ruhr-Universität Bochum, Bochum, Germany; 2 University of Montreal, Hospital Research Center (CRCHUM) and School of Public Health, Montreal, Canada; 3 Dental School, College of Medicine Veterinary and Life Sciences, University of Glasgow, Glasgow, United Kingdom; 4 International Agency for Research on Cancer (IARC), Lyon, France; 5 Institute of Environmental Medicine, Karolinska Institutet, Stockholm, Sweden; 6 Inserm, Centre for Research in Epidemiology and Population Health (CESP), U1018, Environmental Epidemiology of Cancer Team, Villejuif, France; 7 University Paris-Sud, UMRS 1018, Villejuif, France; 8 Institute for Medical Informatics, Biometry and Epidemiology, University Hospital Essen, Essen, Germany; 9 Leibniz-Institute for Prevention Research and Epidemiology -BIPS GmbH, Bremen, Germany; 10 Institute for Statistics, University Bremen, Bremen, Germany; 11 Institute of Epidemiology I, Helmholtz Zentrum München, Neuherberg, Germany; 12 Institute of Medical Statistics and Epidemiology, Technical University Munich, Munich, Germany; 13 Unit of Epidemiology, Fondazione IRCCS Ca' Granda-Ospedale Maggiore Policlinico, Milan, Italy; 14 Unit of Cancer Epidemiology, Department of Medical Sciences, University of Turin, Turin, Italy; 15 Laboratory of Public Health and Population Studies, Department of Molecular Medicine, University of Padova, Padova, Italy; 16 Epidemiology Unit, Istituto Dermopatico dell'Immacolata (IDI-IRCCS-FLMM), Rome, Italy; 17 INRS-Institut Armand-Frappier, Université du Québec, Laval, Québec, Canada; 18 Public Health Ontario, Toronto, Canada; 19 Cancer Care Ontario, Occupational Cancer Research Centre, Toronto, Canada; 20 National Cancer Institute, Division of Cancer Epidemiology and Genetics, Bethesda, United States of America; 21 Molecular Epidemiology of Cancer Unit, University of Oviedo-Ciber de Epidemiologia, CIBERESP, Oviedo, Spain; 22 Institute of Carcinogenesis, Russian Cancer Research Centre, Moscow, Russia; 23 The Nofer Institute of Occupational Medicine, Lodz, Poland; 24 National Centre for Public Health, Budapest, Hungary; 25 The M Sklodowska-Curie Cancer Center and Institute of Oncology, Warsaw, Poland; 26 Regional Authority of Public Health, Preventive Occupational Medicine, Banska Bystrica, Slovakia; 27 Roy Castle Lung Cancer Research Programme, Cancer Research Centre, University of Liverpool, Liverpool, United Kingdom; 28 National Institute of Public Health, Bucharest, Romania; 29 Institute of Hygiene and Epidemiology, 1st Faculty of Medicine, Charles University, Prague, Czech Republic; 30 Masaryk Memorial Cancer Institute and Medical Faculty of Masaryk University, Dept. of Cancer Epidemiology & Genetics, Brno, Czech Republic; 31 Palacky University, Faculty of Medicine, Olomouc, Czech Republic; 32 Department of Epidemiology and Public Health, Faculty of Medicine, University of Ostrava, Ostrava, Czech Republic; 33 Environmental Epidemiology Division, Institute for Risk Assessment Sciences, Utrecht University, Utrecht, The Netherlands; 34 The Tisch Cancer Institute, Icahn School of Medicine at Mount Sinai, New York, New York, United States of America; TNO, NETHERLANDS

## Abstract

**Background:**

An association between low socioeconomic status (SES) and lung cancer has been observed in several studies, but often without adequate control for smoking behavior. We studied the association between lung cancer and occupationally derived SES, using data from the international pooled SYNERGY study.

**Methods:**

Twelve case-control studies from Europe and Canada were included in the analysis. Based on occupational histories of study participants we measured SES using the International Socio-Economic Index of Occupational Status (ISEI) and the European Socio-economic Classification (ESeC). We divided the ISEI range into categories, using various criteria. Stratifying by gender, we calculated odds ratios (OR) and 95% confidence intervals (CI) by unconditional logistic regression, adjusting for age, study, and smoking behavior. We conducted analyses by histological subtypes of lung cancer and subgroup analyses by study region, birth cohort, education and occupational exposure to known lung carcinogens.

**Results:**

The analysis dataset included 17,021 cases and 20,885 controls. There was a strong elevated OR between lung cancer and low SES, which was attenuated substantially after adjustment for smoking, however a social gradient persisted. SES differences in lung cancer risk were higher among men (lowest vs. highest SES category: ISEI OR 1.84 (95% CI 1.61–2.09); ESeC OR 1.53 (95% CI 1.44–1.63)), than among women (lowest vs. highest SES category: ISEI OR 1.54 (95% CI 1.20–1.98); ESeC OR 1.34 (95% CI 1.19–1.52)).

**Conclusion:**

SES remained a risk factor for lung cancer after adjustment for smoking behavior.

## Introduction

Lung cancer has the highest mortality rate of all cancers worldwide [1]. Socioeconomic status (SES) has been associated with lung cancer in several studies, with people from lower socioeconomic backgrounds having the highest incidence rates [2–8]. SES reflects one’s position in societal hierarchies, and is generally assessed by the interdependent dimensions of education, occupation and income. SES is linked with health/disease through multiple interacting pathways in terms of material and social resources, physical and psycho-social stressors, and health-related behaviors [9,10]. SES is strongly associated with smoking behavior [11], the most important risk factor in the etiology of lung cancer. However, many studies on lung cancer and SES do not adequately control for smoking behavior [12], and findings about the extent to what SES is explained by smoking are not consistent [3,7,13,14]. We investigated whether SES is a risk factor for lung cancer, and to what extent the association is reduced by consideration of smoking. We operationalized SES by two different occupation-based concepts. First, we measured SES by application of the International Socio-Economic Index of occupational status (ISEI) [15]. ISEI was originally constructed to create an internationally comparable socio-economic index by combining data on education, income, and occupation as the three main dimensions of SES. The different ISEI scores for occupations were calculated by assuming that occupation represents an intermediate factor which converts education into income [15]. Second, we used the European Socio-economic Classification (ESeC), which categorizes social positions on the basis of typical employment relations and conditions of occupations [16]. We applied these two concepts to different job periods to investigate variations of occupational SES and lung cancer associations. Additionally, we explored whether the relationships between SES and lung cancer differed by histological tumor subtype, and conducted subgroup analyses to explore effects according to study region, occupational exposures, smoking status, education, birth cohort, study control type and city size of last residence. Considering biological as well as social differences between men and women with regard to lung cancer [17], we stratified all analysis by gender.

## Materials and methods

### Data availability

We analyzed data from the SYNERGY study (‘Pooled Analysis of Case-Control Studies on the Joint Effects of Occupational Carcinogens in the Development of Lung Cancer’) database. Detailed information on the SYNERGY project has been published previously [18,19] and is available at the study website (http://synergy.iarc.fr). Briefly, SYNERGY is an international collaboration to study the role of occupational exposures on lung cancer risk. All included studies solicited detailed information on the participants’ occupational biography (ISCO-68 coded job periods along with ISIC (Rev. 2) coded industries) and smoking history. Individual participant data from 16 studies and 22 study centers conducted between 1985 and 2010 are currently included in SYNERGY. The ethics committees of the individual studies approved the conduct of the study, as well as the Institutional Review Board of the International Agency for Research on Cancer. Study subjects or -in the case of deceased subjects- their relatives gave written informed consent to participate in the study.

We included studies from Europe and North America and used data from 12 studies conducted in 18 study centers. We excluded two studies because of missing information: The MORGEN study (Netherlands) did not contain data on the time since smoking cessation for former smokers, and the PARIS study (France) did not have information on education and was restricted to smokers. Participants were excluded if they had no ISCO codes in their occupational history to derive occupational SES (n = 651). These included, for example, housewives, participants working exclusively in the military or lifetime unemployed. Participants with missing smoking history were also excluded (n = 23).

Cases were histologically confirmed lung cancer cases, categorized into lung cancer subtypes (squamous cell carcinoma (SQCC), small cell lung cancer (SCLC), adenocarcinoma (ADC), other/unspecified).

Information was available on several further variables, which either constituted the “exposure variables” or covariates. This included gender, age, geographic area of residence, smoking history, education, and occupational history. The occupational history was used to create the “exposure variables” and to create an indicator of potential exposure to occupational carcinogens.

### Indices of socioeconomic status

In order to classify the SES of study participants, we used two indices that can be assigned by the participant’s occupation, namely, the ISEI [15] and the ESeC [16]. The ISEI is a continuous status score for occupations, derived by Ganzeboom and co-workers based on age, education and income. The minimum score was 10 (e.g. for cook’s helpers), the maximum 90 (judges). We used each participant’s job history in conjunction with the ISEI score for the occupations to assign an ISEI score to each job. We categorized subjects into categories in two ways: first by dividing the entire ISEI range into four equal sub-ranges (10-29, 30-50, 51-70, 71-90 points) and second by calculating frequency distribution quartiles based on the gender-specific distribution of scores among control subjects.

The ESeC is a derivative of the Erikson-Goldthorpe-Portocarero (EGP) scheme [20]. In contrast to the continuous ISEI scale, ESeC defines discrete categories of social positions: Occupations are classified according to their typical employment relations and conditions referring to the labor market (income, security, prospects) and work situation (authority, autonomy) [21]. We applied the ESeC with 3 classes (“The Salariat”, “Intermediate”, and “Working Class”), which shows a hierarchical order unlike the original scale of 9 classes (optionally plus the class of unemployment, which we analyzed independently). The condensed version is recommended by the ESeC-authors when additional information about employment status and size of organization is missing [21].

For the assignment of the indicators we utilized instruments available on the authors’ websites [21, 22]. We assigned scores based on each participant’s longest, first and last held job period and additionally, the lowest and highest score ever reached (ISEI only). Jobless periods due to unemployment (including illness) were assessed separately. We categorized the maximum duration of unemployed periods and, for comparison, the sum of unemployed years for each participant (never, >0–1, >1–5, >5–10, >10 years). We further categorized participants in those who ever or never worked in blue collar jobs by the first digit of ISCO codes (transformed into ISCO-88) (white-collar: 1–5, blue-collar: 6–9).

Education was categorized as follows: no formal/some primary education (<6 years), primary/some secondary education (6–9 years), secondary education/some college (10–13 years), university.

### Covariates

The smoking history was parametrized by means of multiple variables: smoking status (non-smokers, former, current cigarette smokers, and smokers of other types of tobacco only), years since quitting smoking, and pack-years (log(cigarette pack-years+1)). Non-smokers were defined as participants who smoked less than one pack-year. Smokers were considered former smokers if they had quit smoking at least 2 years before the interview/diagnosis; otherwise they were considered current smokers [23]. Former smokers were subdivided into categories of 2–5, 6–10, 11–15, 16–25, 26–35 and more than 35 years since quitting smoking.

To indicate occupational exposures to lung carcinogens, we used a classification of occupations developed by Ahrens and Merletti [24] on the basis of occupational categories (ISCO-68) and industrial sectors (ISIC Rev.2). The list of occupations with potential carcinogenic risk is known as ‘list A’ and includes, among others, jobs in metal production and processing, construction, mining, the chemical industry, asbestos production [24,25]. Participants were classified as ever or never having worked in a ‘list A’ job.

We combined countries to the following study regions: Northern/Central Europe (France, Germany, Sweden, United Kingdom), Eastern Europe (Czech Republic, Hungary, Poland, Romania, Russia, Slovakia), Southern Europe (Italy, Spain), and Canada. We differentiated whether controls were recruited population-based or in hospitals. We categorized birth cohorts (<1930, 1930–1939, >1939) and city size of last residence (rural/midsize: < = 100,000 inhabitants, urban: >100,000 inhabitants).

### Statistical analysis

We estimated odds ratios (OR) with 95% confidence intervals (CI) by unconditional logistic regression models, and used the longest held job for the main analyses. Categories with the highest SES were set as reference. We adjusted for log(age) and study center in model 1 and added smoking variables in model 2. We stratified analyses by gender, restricted in some cases to men because of insufficient numbers in women. We calculated tests for trend for all analyses. To quantify the difference of ORs between the two models, we applied ((OR_model1_–OR_model2_)/(OR_model1_−1)*100) [13, 26].

We additionally adjusted models for educational level as a second SES indicator and ‘list A’ to study the impact on the association of occupational SES and lung cancer.

To investigate whether the SES-lung cancer associations differed by histologic type, we conducted separate analyses in the main histological subtypes of lung cancer (SQCC, SCLC, ADC).

Subgroup or sensitivity analyses were conducted to elucidate possible effects by education, study region, city size of last residence, birth cohort, employment in ‘list A’ job, employed in a blue collar job, smoking status, and type of control recruitment.

We calculated correlations between the selected job periods (first, last, etc.) and correlations with education by Spearman's rank correlation coefficient for ISEI and by Cramér’s V for ESeC.

We used random-effect meta-regression models to examine heterogeneity between study centers. The LUCA study was not included in the meta-analysis because adjustment for smoking was not possible due to missing cases in the reference category (non-smokers).

All statistical analyses were carried out with SAS, version 9.3 (SAS Institute Inc., Cary, NC) except for meta-analyses, which were performed using Comprehensive Meta-Analysis Version 2.2.027 software (Biostat, Englewood, NJ).

## Results

### Characteristics of the study population

Altogether, 17,021 cases of lung cancer and 20,885 controls were included in the final analysis. The characteristics of the study participants are shown in Table 1. Approximately 80% of cases and controls were male. Lung cancer cases less frequently held jobs in the highest occupational categories, had lower education, were more frequently smokers at time of interview, had smoked more pack-years and slightly more often experienced unemployment than controls. Fractions of participants with higher occupational SES (summing up the two upper categories of ISEI and ESeC, respectively), higher education, and non-smokers were lower among men. The maximum duration of periods of unemployment was higher for women than for men.

**Table 1:** Characteristics of the study population by gender and case-control status.

	Men		Women	
Characteristics	Cases n (%)	Controls n (%)	Cases n (%)	Controls n (%)
Age (years)				
Median (Interquartile range)	63 (57–69)	63 (56–69)	61.0 (53–69)	61.0 (52–69)
ISEI (longest job)				
1^st^ quarter (71–90)	591 (4.3)	1482 (9.0)	146 (4.5)	293 (6.7)
2^nd^ quarter (51–70)	2449 (17.8)	4297 (26.1)	1002 (30.8)	1534 (34.8)
3^rd^ quarter (30–50)	8415 (61.1)	8471 (51.4)	1218 (37.5)	1600 (36.3)
4^th^ quarter (10–29)	2317 (16.8)	2230 (13.5)	883 (27.2)	978 (22.2)
ESeC (longest job)				
The Salariat	3262 (23.7)	5517 (33.5)	830 (25.5)	1405 (31.9)
Intermediate	1888 (13.7)	2819 (17.1)	684 (21.1)	950 (21.6)
Working Class	8622 (62.6)	8144 (49.4)	1735 (53.4)	2050 (46.5)
Duration of unemployment (longest period)				
Never	12,125 (88.0)	14,885 (90.3)	2878 (88.6)	3955 (89.8)
>0–1 year	557 (4.0)	618 (3.8)	118 (3.6)	133 (3.0)
>1–5 years	708 (5.1)	684 (4.2)	151 (4.6)	182 (4.1)
>5–10 years	233 (1.7)	164 (1.0)	50 (1.5)	72 (1.6)
>10 years	149 (1.1)	129 (0.8)	52 (1.6)	63 (1.4)
Education				
University	1401 (10.2)	2920 (17.7)	488 (15.0)	913 (20.7)
Secondary/some college (10–13 years)	2568 (18.6)	4095 (24.8)	705 (21.7)	1117 (25.4)
Primary/some secondary (6–9 years)	6600 (47.9)	6861 (41.6)	1417 (43.6)	1577 (35.8)
No formal education/some primary (<6 years)	2736 (19.9)	2326 (14.1)	560 (17.2)	729 (16.5)
Missing	467 (3.4)	278 (1.7)	79 (2.4)	69 (1.6)
Smoking status				
Non-smoker	336 (2.4)	4066 (24.7)	877 (27.0)	2650 (60.2)
Former smoker	4876 (35.4)	7410 (45.0)	641 (19.7)	885 (20.1)
Current smoker	8407 (61.0)	4596 (27.9)	1731 (53.3)	868 (19.7)
Other types of tobacco	153 (1.1)	408 (2.5)	0 (0)	2 (0)
Pack-years				
Median (Interquartile range)	38.5 (25.3–54.0)	14.0 (0–32.1)	22.5 (0–40.0)	0 (0–10.0)
Histological lung tumor subtypes				
SQCC	5866 (42.6)		658 (20.3)	
SCLC	2195 (15.9)		524 (16.1)	
ADC	3424 (24.9)		1409 (43.4)	
Other/unspecified	2207 (16.0)		644 (19.8)	
Missing	80 (0.6)		14 (0.4)	
Birth cohort				
<1930	5328 (38.7)	6523 (39.6)	961 (29.6)	1109 (25.2)
1930–1939	4673 (33.9)	5032 (30.5)	995 (30.6)	1432 (32.5)
>1939	3771 (27.4)	4925 (29.9)	1293 (39.8)	1864 (42.3)
Study region				
Northern/Central Europe	7298 (53.0)	9416 (57.1)	1440 (44.3)	1799 (40.8)
Eastern Europe	2032 (14.8)	1992 (12.1)	560 (17.2)	670 (15.2)
Southern Europe	3536 (25.7)	3818 (23.2)	621 (19.1)	860 (19.5)
Canada	906 (6.6)	1254 (7.6)	628 (19.3)	1076 (24.4)
Ever worked in list-A job				
Yes	2056 (14.9)	1570 (9.5)	85 (2.6)	55 (1.2)
No	11,716 (85.1)	14,910 (90.5)	3164 (97.4)	4350 (98.8)
Ever worked in blue-collar job				
Yes	11,315 (82.2)	11,899 (72.2)	1872 (57.6)	2289 (52.0)
No	2457 (17.8)	4581 (27.8)	1377 (42.4)	2116 (48.0)
Size of last residence				
Urban (>100,000)	6470 (47.0)	7812 (47.4)	1674 (51.5)	2044 (46.4)
Rural/midsize (< = 100,000)	4303 (31.2)	4639 (28.1)	665 (20.5)	819 (18.6)
Missing	2999 (21.8)	4029 (24.4)	910 (28.0)	1542 (35.0)
Total	13,772 (36.3)	16,480 (43.5)	3249 (8.6)	4405 (11.6)

When combining the upper categories of ISEI to high SES and the lower categories to low SES, current smokers represented 47% of men and 36% of women with low SES compared to 34% of men and 31% of women with high SES. Non-smokers accounted for 12% of men with high SES and 20% of men with low SES. In women, the proportion of non-smokers was equal for low and high SES (46%).

The distribution of SES among the controls varied by study center in particular with a higher proportion of lower SES in CAPUA (Spain) and higher SES in TORONTO (Canada) (S1A Fig and S1B Fig).

### Associations between SES and lung cancer

Table 2 displays the association of occupational SES, applied to the longest held job, and lung cancer, comparing models with and without adjustment for smoking. Risk estimates increased as SES decreased. Adjustment for smoking behavior decreased the ORs, but elevated ORs between SES and lung cancer remained even after adjustment for smoking. The effect of SES was greater among men than among women. These observations generally applied to all types of selected job periods of ISEI and ESeC, with corresponding tests for trend (S1 and S2 Tables). The average reduction due to adjustment for smoking habits in men was 50% for ISEI and 26% for ESeC, and in women 34% for ISEI and 9% for ESeC. Unemployment with a maximum duration of >5–10 years and >10 years was associated with an increased risk of lung cancer for men (Table 3). Similar results were observed for cumulative unemployment of 5–10 years and > 10 years (S3 Table).

**Table 2:** Estimated lung cancer risks (OR) with 95% confidence intervals (CI) for occupational SES (ISEI^a^ and ESeC of the longest job) by gender.

SES–gender	Cases		Controls		Model 1^b^	Model 2^c^
	n	%	n	%	OR (95%-CI)	OR (95%-CI)
ISEI–men						
1^st^ quarter (71–90)	591	4.3	1482	9.0	1.00	1.00
2^nd^ quarter (51–70)	2449	17.8	4297	26.1	1.42 (1.28–1.59)	1.18 (1.04–1.34)
3^rd^ quarter (30–50)	8415	61.1	8471	51.4	2.49 (2.25–2.76)	1.80 (1.60–2.02)
4^th^ quarter (10–29)	2317	16.8	2230	13.5	2.59 (2.31–2.90)	1.84 (1.61–2.09)
Test for trend					P < 0.001	P < 0.001
ISEI–women						
1^st^ quarter (71–90)	146	4.5	293	6.7	1.00	1.00
2^nd^ quarter (51–70)	1002	30.8	1534	34.8	1.27 (1.02–1.58)	1.16 (0.91–1.48)
3^rd^ quarter (30–50)	1218	37.5	1600	36.3	1.44 (1.16–1.79)	1.28 (1.00–1.63)
4^th^ quarter (10–29)	883	27.2	978	22.2	1.72 (1.37–2.15)	1.54 (1.20–1.98)
Test for trend					P < 0.001	P < 0.001
ESeC–men						
The Salariat	3262	23.7	5517	33.5	1.00	1.00
Intermediate	1888	13.7	2819	17.1	1.10 (1.02–1.18)	1.08 (0.99–1.17)
Working Class	8622	62.6	8144	49.4	1.79 (1.70–1.89)	1.53 (1.44–1.63)
Test for trend					P < 0.001	P < 0.001
ESeC–women						
The Salariat	830	25.5	1405	31.9	1.00	1.00
Intermediate	684	21.1	950	21.6	1.22 (1.07–1.40)	1.22 (1.05–1.42)
Working Class	1735	53.4	2050	46.5	1.41 (1.27–1.58)	1.34 (1.19–1.52)
Test for trend					P < 0.001	P < 0.001

^a^ Categories by quarters of ISEI range.

^b^ Adjusted for log(age) and study center.

^c^ Adjusted for log(age), study center, smoking status incl. time since quitting (current smoker, quitted 2–5, 6–10, 11–15, 16–25, 26–35 or >35 years before interview/diagnosis, only other types of tobacco, non-smoker) and cigarette pack-years (log(py+1)).

**Table 3:** Estimated lung cancer risks (OR) with 95% confidence intervals (CI) for categories of the longest period of unemployment by gender.

	Cases		Controls		Model 1^a^	Model 2^b^
Duration of unemployment	n	%	n	%	OR (95%-CI)	OR (95%-CI)
Men						
Never unemployed	12125	88.0	14885	90.3	1.00	1.00
>0–1 year	557	4.0	618	3.8	1.07 (0.94–1.20)	1.01 (0.88–1.15)
>1–5 years	708	5.1	684	4.2	1.21 (1.09–1.36)	1.02 (0.90–1.16)
>5–10 years	233	1.7	164	1.0	1.76 (1.43–2.16)	1.40 (1.11–1.76)
>10 years	149	1.1	129	0.8	1.57 (1.23–2.00)	1.21 (0.92–1.60)
Test for trend					P < 0.001	P = 0.022
Women						
Never unemployed	2878	88.6	3955	89.8	1.00	1.00
>0–1 year	118	3.6	133	3.0	1.12 (0.86–1.45)	0.91 (0.67–1.22)
>1–5 years	151	4.6	182	4.1	1.04 (0.83–1.31)	0.97 (0.75–1.25)
>5–10 years	50	1.5	72	1.6	0.93 (0.64–1.35)	0.80 (0.52–1.21)
>10 years	52	1.6	63	1.4	1.18 (0.81–1.72)	0.90 (0.59–1.37)
Test for trend					P = 0.487	P = 0.291

^a^ Adjusted for log(age) and study center.

^b^ Adjusted for log(age), study center, smoking status incl. time since quitting (current smoker, quitted 2–5, 6–10, 11–15, 16–25, 26–35 or >35 years before interview/diagnosis, only other types of tobacco, non-smoker) and cigarette pack-years (log(py+1)).

The results for either ISEI categorization, based on the score-range or the gender-specific control distribution (S4 Table), showed similar ORs. We also observed similar associations between SES and lung cancer for the longest and last job periods and the highest ever reached ISEI on the one hand, and for the first job and the lowest ever reached ISEI on the other hand. The job periods within these two groups (longest job/last job/highest ISEI and first job/lowest ISEI, respectively) were highly correlated (S5 Table). Additional adjustment for education further reduced risk estimates on average by approximately 50% whereas adjustment for ‘list A’ resulted in a slight reduction (S6 Table). Occupational SES correlated moderately with education (ISEI–Spearman’s r 0.45, ESeC–Cramér’s V 0.31).

When stratifying the data by histological tumor subtype (Table 4), we observed increased ORs for SQCC and SCLC and slightly reduced risks for the lower SES-categories for ADC. In women, adjustment for smoking behavior increased ORs for SQCC and SCLC in the lower SES categories.

**Table 4:** Association of SES (ISEI^a^ –longest job) and lung cancer by histological tumor subtype.

Tumor subtypes–gender	Controls	Cases	Model 1^b^ OR (95%-CI)	Model 2^c^ OR (95%-CI)
Squamous Cell Carcinoma–men				
1^st^ quarter (71–90)	1482	205	1.00	1.00
2^nd^ quarter (51–70)	4297	921	1.56 (1.33–1.84)	1.30 (1.09–1.56)
3^rd^ quarter (30–50)	8471	3645	3.11 (2.67–3.63)	2.25 (1.90–2.66)
4^th^ quarter (10–29)	2230	1095	3.55 (3.00–4.19)	2.53 (2.11–3.04)
Test for trend			P < 0.001	P < 0.001
Squamous Cell Carcinoma–women				
1^st^ quarter (71–90)	293	23	1.00	1.00
2^nd^ quarter (51–70)	1534	174	1.36 (0.86–2.16)	1.39 (0.83–2.34)
3^rd^ quarter (30–50)	1600	267	1.81 (1.15–2.84)	1.86 (1.11–3.09)
4^th^ quarter (10–29)	978	194	2.11 (1.33–3.35)	2.50 (1.48–4.22)
Test for trend			P < 0.001	P < 0.001
Small Cell Lung Cancer–men				
1^st^ quarter (71–90)	1482	84	1.00	1.00
2^nd^ quarter (51–70)	4297	365	1.58 (1.24–2.03)	1.30 (1.00–1.68)
3^rd^ quarter (30–50)	8471	1366	3.03 (2.41–3.81)	2.12 (1.66–2.70)
4^th^ quarter (10–29)	2230	380	3.18 (2.48–4.08)	2.13 (1.63–2.77)
Test for trend			P < 0.001	P < 0.001
Small Cell Lung Cancer–women				
1^st^ quarter (71–90)	293	17	1.00	1.00
2^nd^ quarter (51–70)	1534	146	1.54 (0.91–2.60)	1.57 (0.87–2.83)
3^rd^ quarter (30–50)	1600	204	1.93 (1.15–3.25)	1.84 (1.03–3.30)
4^th^ quarter (10–29)	978	157	2.54 (1.50–4.30)	2.85 (1.57–5.18)
Test for trend			P < 0.001	P < 0.001
Adenocarcinoma–men				
1^st^ quarter (71–90)	1482	188	1.00	1.00
2^nd^ quarter (51–70)	4297	755	1.34 (1.13–1.59)	1.15 (0.96–1.38)
3^rd^ quarter (30–50)	8471	2011	1.83 (1.55–2.15)	1.38 (1.16–1.64)
4^th^ quarter (10–29)	2230	470	1.57 (1.31–1.89)	1.17 (0.96–1.42)
Test for trend			P < 0.001	P = 0.009
Adenocarcinoma–women				
1^st^ quarter (71–90)	293	76	1.00	1.00
2^nd^ quarter (51–70)	1534	460	1.10 (0.83–1.45)	1.02 (0.76–1.38)
3^rd^ quarter (30–50)	1600	511	1.17 (0.89–1.54)	1.06 (0.79–1.42)
4^th^ quarter (10–29)	978	362	1.32 (0.99–1.77)	1.24 (0.91–1.68)
Test for trend			P = 0.012	P = 0.039

^a^ Categories by quarters of ISEI range.

^b^ Odds ratio with 95% confidence interval–adjusted for log(age), study center.

^c^ Odds ratio with 95% confidence interval–adjusted for log(age), study center, smoking status incl. time since quitting (current smoker, quitted 2–5, 6–10, 11–15, 16–25, 26–35 or >35 years before interview/diagnosis, only other types of tobacco, non-smoker) and cigarette pack-years (log(py+1)).

### Subgroup and sensitivity analyses, meta-analysis

Table 5 shows results for the subgroup analyses: The effect estimates remained unchanged for participants who never or ever worked in a ‘list A’ job and for male non-smokers of the lowest SES category. ORs were comparatively higher for population than hospital controls; lower for participants most recently residing in an urban area, and also for men who never held a blue-collar job. When exploring last residence in urban area for the younger half of the study population (< 63 years) ORs increased marginally for women (S7 Table).

**Table 5:** Association of SES (ISEI^a^ –longest job) and lung cancer in subgroups.

	Men			Women		
Subgroup	Cases	Controls	OR (95%-CI)^b^	Cases	Controls	OR (95%-CI)^b^
Never worked in a List-A occupation						
1st quarter (71–90)	567	1447	1.00	144	293	1.00
2nd quarter (51–70)	2312	4130	1.18 (1.04–1.34)	994	1527	1.17 (0.92–1.50)
3rd quarter (30–50)	6932	7408	1.74 (1.55–1.96)	1181	1575	1.28 (1.00–1.63)
4th quarter (10–29)	1905	1925	1.77 (1.55–2.03)	845	955	1.53 (1.18–1.97)
Test for trend			P < 0.001			P < 0.001
Ever worked in a List-A occupation						
1st quarter (71–90)	24	35	1.00	2	0	
2nd quarter (51–70)	137	167	1.12 (0.59–2.10)	8	7	
3rd quarter (30–50)	1483	1063	1.79 (0.99–3.22)	37	25	
4th quarter (10–29)	412	305	1.79 (0.98–3.28)	38	23	
Test for trend			P = 0.005			
Never worked in a blue-collar occupation						
1st quarter (71–90)	365	1016	1.00	126	254	1.00
2nd quarter (51–70)	1292	2457	1.10 (0.94–1.29)	771	1211	1.21 (0.93–1.58)
3rd quarter (30–50)	726	1014	1.44 (1.21–1.73)	414	599	1.25 (0.94–1.66)
4th quarter (10–29)	74	94	1.45 (0.99–2.13)	66	52	2.15 (1.32–3.48)
Test for trend			P < 0.001			P = 0.051
Ever worked in a blue-collar occupation						
1st quarter (71–90)	226	466	1.00	20	39	1.00
2nd quarter (51–70)	1157	1840	1.25 (1.03–1.53)	231	323	1.04 (0.55–1.96)
3rd quarter (30–50)	7689	7457	1.87 (1.56–2.25)	804	1001	1.29 (0.70–2.39)
4th quarter (10–29)	2243	2136	1.89 (1.56–2.29)	817	926	1.51 (0.82–2.80)
Test for trend			P < 0.001			P = 0.002
Population controls						
1st quarter (71–90)	476	1272	1.00	105	201	1.00
2nd quarter (51–70)	2128	3708	1.24 (1.08–1.42)	910	1208	1.41 (1.06–1.87)
3rd quarter (30–50)	6732	6655	2.01 (1.76–2.28)	1049	1230	1.59 (1.19–2.12)
4th quarter (10–29)	1880	1703	2.14 (1.85–2.47)	790	775	1.99 (1.48–2.67)
Test for trend			P < 0.001			P < 0.001
Hospital controls						
1st quarter (71–90)	160	200	1.00	77	88	1.00
2nd quarter (51–70)	467	528	0.97 (0.73–1.28)	276	312	0.81 (0.55–1.20)
3rd quarter (30–50)	2115	1688	1.19 (0.92–1.53)	319	342	0.78 (0.52–1.15)
4th quarter (10–29)	554	464	1.11 (0.84–1.47)	162	186	0.74 (0.48–1.13)
Test for trend			P = 0.119			P = 0.217
Non-smokers			^c^			^c^
1st quarter (71–90)	32	470	1.00	56	181	1.00
2nd quarter (51–70)	81	1146	1.02 (0.66–1.56)	262	870	1.04 (0.74–1.47)
3rd quarter (30–50)	164	1965	1.36 (0.91–2.03)	340	932	1.17 (0.83–1.64)
4th quarter (10–29)	59	485	1.88 (1.19–2.98)	219	667	1.06 (0.74–1.51)
Test for trend			P < 0.001			P = 0.641
Urban last residence (>100,000 inhabitants)						
1st quarter (71–90)	302	697	1.00	81	121	1.00
2nd quarter (51–70)	1313	2166	1.16 (0.98–1.38)	542	738	0.87 (0.61–1.24)
3rd quarter (30–50)	3893	3953	1.60 (1.37–1.88)	612	758	0.90 (0.64–1.28)
4th quarter (10–29)	962	996	1.49 (1.24–1.79)	439	427	1.15 (0.80–1.66)
Test for trend			P < 0.001			P = 0.046

^a^ Categories by quarters of ISEI range.

^b^ Odds ratio with 95% confidence interval–adjusted for log(age), study center, smoking status incl. time since quitting (current smoker, quitted 2–5, 6–10, 11–15, 16–25, 26–35 or >35 years before interview/diagnosis, only other types of tobacco, non-smoker) and cigarette pack-years (log(py+1)).

^c^ Odds ratio with 95% confidence interval–adjusted for log(age), study center and cigarette pack-years (log(py+1)).

Stratification by study region (S8 Table) revealed higher ORs in Northern/Central Europe and lower ORs in the other regions with a negative association for women in Eastern Europe. In comparison to the score-based categorization of ISEI, applying gender-specific ISEI-quartiles attenuated associations for women except for Canada, and increased ORs in men for Southern Europe. ORs increased in the birth cohort of 1930–1939 for men and, especially in the middle SES categories, in the birth cohort >1939 for women (S9 Table). The lung cancer risk of the lower SES-groups decreased when stratifying for education, especially in the strata of higher education (S10 Table).

Meta-analyses (S2 Fig) showed slightly lower overall ORs than the corresponding pooled ORs. The stronger the association of lung cancer and SES, the higher were the proportions of heterogeneity with above 60% for at least the lowest vs. highest SES-categories.

## Discussion

In this study we confirmed a social gradient for lung cancer, with greater risk associated with lower occupational SES that persisted after adjustment for smoking habits and was higher among men. Smoking habits reduced only up to half of the lung cancer risk of lower SES. Additional adjustment for education further (but not completely) attenuated the ORs. Despite regional differences, lung cancer risks were still elevated especially for the lowest SES categories with exception of women in Eastern Europe. Unemployment was not associated with lung cancer except for subjects who experienced unemployed periods >5 years, and this finding was restricted to men.

Strengths of this study are primarily based on the large international SYNERGY database with participants’ detailed occupational and smoking histories. Smoking information was nearly complete, which allowed for a detailed control of smoking behavior, as recommended in the literature [14]. The ISCO-coded job biographies permitted the assignment of international validated SES indicators to nearly the entire dataset (98%).

Limitations include the validity of the SES indicators: ISEI was developed based on data restricted to men. ESeC was developed for comparisons of European countries. Additionally, ISEI and ESeC are occupational indicators restricted to gainfully employed subjects. Even though we analyzed the influence of being unemployed due to loss of job or periods of illness, we could have missed possible influences of activities outside of the workforce, such as housework, part-time work, retirement, which could have underestimated socioeconomic differences [27]. This concerns not only non-occupationally active periods, but also participants without any gainful employment in their job history who were excluded from the analysis. Unfortunately, for lifetime housewives we did not have information on the husband’s occupation for derivation of the SES. We also could have missed effects of early retirement as a hidden form of unemployment. Even though our classification of education was based on an international classification, it generally remains problematic to capture the country-specific implications of time spent in the educational system and corresponding educational attainment.

Another limitation concerns residual effects of smoking behavior due to misclassification: Stratification by histological subtypes revealed higher SES risks for the smoking-associated subtypes (SCLC, SQCC) and reduced SES risks for ADC, which is the histological subtype of lung cancer showing the weakest association with smoking [19]. Furthermore, regional differences as well as elevated risks in the younger female birth cohort in our study correspond to the international patterns of the international ‘smoking epidemic’ observed with regard to SES and lung cancer [6]. The ‘smoking epidemic’ describes the historical prevalence of smoking that differed by countries/regions (e.g. Northern compared with Southern Europe), gender, and SES [28]. We identified elevated risks for male non-smokers, which could be due to our definition of non-smokers (<1 cigarette pack-year) that also includes occasional smokers. Measuring smoking in pack-years as cumulative lifetime dose may underestimate the role of smoking duration in relation to smoking intensity [29]. Despite evidence for the accuracy of self-reported smoking habits across various occupations and industries [30], recall bias and differential misclassification of smoking cannot be ruled out. Given the several indications and possibilities for residual effects of smoking, we assume that we rather overestimated the effects of SES on lung cancer.

Third, the possibility of selection bias was implied in our analysis because the association between lung cancer and SES was stronger among population than hospital controls. In population-based studies subjects of lower SES tend to show lower participation [31], and case-control studies on lung cancer and SES with population-based controls revealed higher ORs for low SES [12]. SES-related non-response bias, i.e. less participation of cases with high SES and of controls with low SES, was observed in one study which was also included in SYNERGY [32]. However, in our study hospital-based recruitment was mainly done in study centers from Eastern Europe making it difficult to distinguish between region-specific and recruitment-based effects.

Further limitations include that we did not have information on other risk factors for lung cancer, e.g. environmental tobacco smoke (ETS) [33] or residential air pollution [34]. We analyzed the city size of the last residence as a proxy for air pollution, but in contrast to the assumption of increased associations in more urban areas, we found risk estimates to be reduced. This also included the subgroup of participants < 63 years of age, indicating the absence of a ‘mobility’ effect among senior citizens. Potential confounders of the association between smoking and lung cancer, which we did not include (e.g. family history of lung cancer) could have also affected our results in terms of mediator-outcome confounding [35].

An important fraction of lung cancer has been attributed to occupational carcinogens [36], but their role in explaining the association of SES and lung cancer has not been fully disentangled yet [4,37]. We considered occupational risk factors by adjustment for ‘list A’ occupations and, alternatively, by excluding participants never working in a ‘list A’ job and did not identify strong differences in the association between SES and lung cancer between these subgroups (Table 5). However, ‘list A’ only lists jobs with a possible exposure to occupational carcinogens and does not include information about exposure probability, intensity, or duration. Blue-collar jobs may include occupational exposures which are not included in ‘list A’. In contrast to subgroup analyses by ‘list A’ occupation, we found slightly higher risk estimates for low SES among ever blue collar as compared to workers never employed in a blue collar job. However, blue collar workers also include participants who were not exposed to occupational carcinogens.

Finally, the applied concept of SES reflects a variety of health-related circumstances and behaviors, but disregards inconsistencies as well as changes of status. Indeed, we recently analyzed social mobility based on occupational prestige in SYNERGY and observed slightly increased associations between lung cancer and downward prestige trajectories over the work life [38]. Here, we measured SES on the individual level with historical information on occupation and additionally education, but extended concepts of SES should involve the entire life course [39], and include income/wealth and area-based measures [40].

We found that adjustment for smoking reduced estimates for the association between SES and lung cancer by up to 50%. This is similar to the findings of Scottish [3], Dutch [41], and European studies [13], and the results for men in a study from Eastern Europe and the UK [7]. In contrast, in a Canadian study the association between SES and lung cancer disappeared after fully adjusting for smoking habits [14]. In our study, the remaining risk estimates were comparatively higher than in most studies on occupational SES and lung cancer, but similar after adjustment for education [12]. However, we focused on the results without education to avoid over adjustment as education is an indicator of SES in early life that remains stable and determines the following SES indicators such as occupation and income [42]. The extent of reduction of ORs due to adjustment for smoking was distinctly lower when we applied ESeC as compared to ISEI. This could point to the different underlying concepts of SES, implying different exposures and pathways to lung cancer. Additionally, ESeC–especially in the condensed version we applied–as well as ISEI categorize ISCO-codes which comprise a hierarchy of occupational skill levels. Applying three ESeC categories may therefore have led to dilution of effects in comparison to the four ISEI categories. A subsequent possible attenuation between SES categories may also have attenuated the effects of smoking in the ESEC categories.

Our analysis of occupational SES was primarily based on the participants’ longest held job, which might reflect durations of possible exposures. As the longest job was highly correlated with the last job, and associations with lung cancer were even slightly elevated–in contrast to the first job–, the last job might be an appropriate choice in similar studies lacking complete occupational histories. The lung cancer risk we found for unemployed men (ever unemployed >1 year, S11 Table) was nearly equal to a large study in five Nordic populations [43], which did not control for smoking behaviors. The observed gender differences in the association of unemployment and lung cancer point to different careers patterns of men and women. Our data confirmed the trend of an increased proportion of ADC at the expense of SQCC and SCLC, when comparing diagnosis before and since the year 2000 (10% more ADC in women, 12% for men), and our analysis of histological lung cancer subtypes supported previous findings, which showed that lung cancer risks for low SES were lower for ADC than for SQCC [6] or SQCC and SCLC [8].

Socioeconomic inequalities in cancer incidence are greatest for lung cancer [8] and our study shows that these inequalities were not explained by smoking behavior. To explain the observed excess risk of lower SES groups, approximately 60% of female non-smokers of the two lower ISEI categories would have had to be misclassified as current smokers with corresponding pack-years. However, assuming 90% of misclassification for men, an OR of approximately 1.5 would have remained for low SES. When we additionally classified former as current smokers, still an OR of 1.2 persisted for low SES. This confirms the need to explore the pathways from SES to lung cancer. First, the effect of exposures to occupational carcinogens via job based SES on lung cancer needs to be further studied. Despite minor effects when considering ‘list A’ jobs in this study, occupational SES directly reflects occupational hazards. Most occupations, such as workers in asbestos production or truck drivers, for which elevated lung cancer risks were demonstrated, were assigned to low SES. As these occupations were traditionally held by men, they may account for the higher ORs for (non-smoking) men in this study. This is supported by the reduced ORs for men who never worked in blue-collar jobs. Further, ETS is also a work-related risk factor for lung cancer [44] and could be particularly linked to occupational SES, as smoking prevalence is higher in lower SES groups.

Other possible, more speculative pathways can be derived from the association of SES and health in general, because occupational and other SES indicators, mainly education and income/wealth, are interdependent. As shown e.g. for education [45], faster biological aging may be associated with low SES.

## Conclusion

Our study showed a persistent SES gradient for lung cancer, even after adjusting for smoking behavior and education. There was some evidence for residual effects of smoking due to misclassification, and at least a part of the regional variance of the association of SES and lung cancer may be explained by these residual effects. Still, the strong associations we found in this study in particular for men emphasize the continuing need for the exploration of the pathways from SES to lung cancer. Clarifying these pathways could then contribute to further understanding of lung cancer etiology and shape prevention approaches.

## Supporting information

S1 Table Estimated lung cancer risks (OR) with 95% confidence intervals (CI) for ISEI categories based on quarters of the score range. (DOCX)

S2 Table Estimated lung cancer risks (OR) with 95% confidence intervals (CI) for ESeC categories. (DOCX)

S3 Table Estimated lung cancer risks (OR) with 95% confidence intervals (CI) for categories of sums of unemployed years. (DOCX)

S4 Table Estimated lung cancer risks (OR) with 95% confidence intervals (CI) for ISEI categories based on gender-specific quartiles of the distribution of the controls. (DOCX)

S5 Table Contains the following: Table A. Correlation of ISEI job periods by Spearman's rank correlation coefficient. Table B. Correlation of ESeC job periods.
(DOCX)

S6 Table Estimated lung cancer risks (OR) with 95% confidence intervals (CI) for occupational SES by gender–with additional adjustment for education or ‘list A’. (DOCX)

S7 Table Association of SES (ISEI–longest job) and lung cancer in participants with last residence in an urban area and age < 63 years. (DOCX)

S8 Table Association of SES (ISEI–longest job) and lung cancer by study region. (DOCX)

S9 Table Association of SES (ISEI–longest job) and lung cancer by birth cohort. (DOCX)

S10 Table Association of SES (ISEI–longest job) and lung cancer by education. (DOCX)

S11 Table Estimated lung cancer risks (OR) with 95% confidence intervals (CI) for unemployment of more than 1 year. (DOCX)

S1 Fig Contains the following: S1A Fig. Distribution of ISEI in male controls by study center. S1B Fig. Distribution of ISEI in female controls by study center.
(DOCX)

S2 Fig Forest plot of odds ratios by study center. (DOCX)

## References

[1] GLOBOCAN 2012: Estimated Cancer Incidence, Mortality and Prevalence Worldwide in 2012 [Internet]: International Agency for Research on Cancer [cited 2015 Jul 27]. Available from: URL:http://globocan.iarc.fr/Default.aspx.

[2] Ekberg-Aronsson M, Nilsson PM, Nilsson J, Pehrsson K, Löfdahl C. Socio-economic status and lung cancer risk including histologic subtyping—A longitudinal study. Lung Cancer 2006; 51(1):21–9. doi: 10.1016/j.lungcan.2005.08.014 16337709 10.1016/j.lungcan.2005.08.014

[3] Hart CL, Hole DJ, Gillis CR, Davey Smith G, Watt GC, Hawthorne VM. Social class differences in lung cancer mortality: risk factor explanations using two Scottish cohort studies. Int J Epidemiol 2001; 30(2):268–74. 11369726 10.1093/ije/30.2.268

[4] Mao Y, Hu J, Ugnat AM, Semenciw R, Fincham S. Socioeconomic status and lung cancer risk in Canada. Int J Epidemiol 2001; 30(4):809–17. 11511609 10.1093/ije/30.4.809

[5] Clegg LX, Reichman ME, Miller BA, Hankey BF, Singh GK, Lin YD, et al Impact of socioeconomic status on cancer incidence and stage at diagnosis: selected findings from the surveillance, epidemiology, and end results: National Longitudinal Mortality Study. Cancer Causes Control 2009; 20(4):417–35. doi: 10.1007/s10552-008-9256-0 19002764 10.1007/s10552-008-9256-0 PMC2711979

[6] Van der Heyden J H A, Schaap MM, Kunst AE, Esnaola S, Borrell C, Cox B, et al Socioeconomic inequalities in lung cancer mortality in 16 European populations. Lung Cancer 2009; 63(3):322–30. doi: 10.1016/j.lungcan.2008.06.006 18656277 10.1016/j.lungcan.2008.06.006

[7] Hruba F, Fabianova E, Bencko V, Cassidy A, Lissowska J, Mates D, et al Socioeconomic indicators and risk of lung cancer in Central and Eastern Europe. Cent Eur J Public Health 2009; 17(3):115–21. 20020599 10.21101/cejph.a3516

[8] Sharpe KH, McMahon AD, McClements P, Watling C, Brewster DH, Conway DI. Socioeconomic inequalities in incidence of lung and upper aero-digestive tract cancer by age, tumor subtype and sex: a population-based study in Scotland (2000–2007). Cancer Epidemiol 2012; 36(3):e164–70. doi: 10.1016/j.canep.2012.01.007 22436397 10.1016/j.canep.2012.01.007

[9] Braveman P, Egerter S, Williams DR. The Social Determinants of Health: Coming of Age. Annu Rev Public Health 2011; 32(1):381–98. 21091195 10.1146/annurev-publhealth-031210-101218

[10] Adler NE, Ostrove JM. Socioeconomic status and health: what we know and what we don’t. Ann N Y Acad Sci 1999; 896:3–15. 10681884 10.1111/j.1749-6632.1999.tb08101.x

[11] Schaap MM, van Agt HM, Kunst AE. Identification of socioeconomic groups at increased risk for smoking in European countries: Looking beyond educational level. Nicotine & tobacco research: official journal of the Society for Research on Nicotine and Tobacco 2008; 10(2):359–69. 18236301 10.1080/14622200701825098

[12] Sidorchuk A, Agardh EE, Aremu O, Hallqvist J, Allebeck P, Moradi T. Socioeconomic differences in lung cancer incidence: a systematic review and meta-analysis. Cancer Causes Control 2009; 20(4):459–71. doi: 10.1007/s10552-009-9300-8 19184626 10.1007/s10552-009-9300-8

[13] Menvielle G, Boshuizen H, Kunst AE, Dalton SO, Vineis P, Bergmann MM, et al The role of smoking and diet in explaining educational inequalities in lung cancer incidence. J Natl Cancer Inst 2009; 101(5):321–30. doi: 10.1093/jnci/djn513 19244178 10.1093/jnci/djn513 PMC2852413

[14] Nkosi TM, Parent M, Siemiatycki J, Rousseau M. Socioeconomic position and lung cancer risk: how important is the modeling of smoking? Epidemiology 2012; 23(3):377–85. doi: 10.1097/EDE.0b013e31824d0548 22415109 10.1097/EDE.0b013e31824d0548

[15] Ganzeboom HB, de Graaf PM, Treiman DJ. A standard international socio-economic index of occupational status. Soc Sci Res 1992; 21(1):1–56.

[16] Rose D, Harrison E. The European Socio-economic Classification: A New Social Class Schema For Comparative European Research. Eur Soc 2007; 9(3):459–90.

[17] Thomas L, Doyle LA, Edelman MJ. Lung cancer in women: emerging differences in epidemiology, biology, and therapy. Chest 2005; 128(1):370–81. doi: 10.1378/chest.128.1.370 16002959 10.1378/chest.128.1.370

[18] Olsson AC, Gustavsson P, Kromhout H, Peters S, Vermeulen R, Brüske I, et al Exposure to diesel motor exhaust and lung cancer risk in a pooled analysis from case-control studies in Europe and Canada. Am J Respir Crit Care Med 2011; 183(7):941–8. doi: 10.1164/rccm.201006-0940OC 21037020 10.1164/rccm.201006-0940OC PMC7259806

[19] Pesch B, Kendzia B, Gustavsson P, Jöckel K, Johnen G, Pohlabeln H, et al Cigarette smoking and lung cancer—relative risk estimates for the major histological types from a pooled analysis of case-control studies. Int J Cancer 2012; 131(5):1210–9. doi: 10.1002/ijc.27339 22052329 10.1002/ijc.27339 PMC3296911

[20] Erikson R, Goldthorpe JH, Portocarero L. Intergenerational class mobility in three Western European societies: England, France and Sweden. Br J Sociol 1979; 30(4):415–41. 10.1111/j.1468-4446.2009.01246.x 20092493

[21] Harrison E, Rose D. The European Socio-economic Classification (ESeC) User Guide: Institute for Social and Economic Research, University of Essex (ISER); 2006 [cited 2018 Jan 4]. Available from: URL:https://www.iser.essex.ac.uk/files/esec/guide/docs/UserGuide.pdf.

[22] Ganzeboom HBG, Treiman DJ. International Stratification and Mobility File: Conversion Tools.: Department of Social Research Methodology; 2012 [cited 2018 Jan 4]. Available from: URL:http://www.harryganzeboom.nl/ismf/index.htm.

[23] Leffondre K, Abrahamowicz M, Xiao Y, Siemiatycki J. Modelling smoking history using a comprehensive smoking index: application to lung cancer. Stat Med 2006; 25(24):4132–46. doi: 10.1002/sim.2680 16998807 10.1002/sim.2680

[24] Ahrens W, Merletti F. A standard tool for the analysis of occupational lung cancer in epidemiologic studies. Int J Occup Environ Health 1998; 4(4):236–40. doi: 10.1179/oeh.1998.4.4.236 9876632 10.1179/oeh.1998.4.4.236

[25] Mirabelli D, Chiusolo M, Calisti R, Massacesi S, Richiardi L, Nesti M, Merletti F. Database of occupations and industrial activities that involve the risk of pulmonary tumors. Epidemiol Prev 2001; 25(4–5):215–21. 11789462

[26] Boing AF, Antunes JL, Carvalho MB de, Góis Filho JF de, Kowalski LP, Michaluart P, et al How much do smoking and alcohol consumption explain socioeconomic inequalities in head and neck cancer risk? J Epidemiol Community Health 2011; 65(8):709–14. doi: 10.1136/jech.2009.097691 20724282 10.1136/jech.2009.097691

[27] Martikainen P, Valkonen T. Bias related to the exclusion of the economically inactive in studies on social class differences in mortality. Int J Epidemiol 1999; 28(5):899–904. 10597989 10.1093/ije/28.5.899

[28] Lopez AD, Collishaw NE, Piha T. A descriptive model of the cigarette epidemic in developed countries. Tob Control 1994; 3(3):242–7.

[29] Peto J. That the effects of smoking should be measured in pack-years: misconceptions 4. Br J Cancer 2012; 107(3):406–7. doi: 10.1038/bjc.2012.97 22828655 10.1038/bjc.2012.97 PMC3405232

[30] Arheart KL, Lee DJ, Fleming LE, LeBlanc WG, Dietz NA, McCollister KE, et al Accuracy of self-reported smoking and secondhand smoke exposure in the US workforce: the National Health and Nutrition Examination Surveys. J Occup Environ Med 2008; 50(12):1414–20. doi: 10.1097/JOM.0b013e318188b90a 19092497 10.1097/JOM.0b013e318188b90a

[31] Conway DI, McMahon AD, Smith K, Taylor JC, McKinney PA. Socioeconomic factors influence selection and participation in a population-based case-control study of head and neck cancer in Scotland. J Clin Epidemiol 2008; 61(11):1187–93. doi: 10.1016/j.jclinepi.2007.12.012 18619798 10.1016/j.jclinepi.2007.12.012

[32] Richiardi L, Boffetta P, Merletti F. Analysis of nonresponse bias in a population-based case–control study on lung cancer. J Clin Epidemiol 2002; 55(10):1033–40. 12464380 10.1016/s0895-4356(02)00455-9

[33] Brennan P, Buffler PA, Reynolds P, Wu AH, Wichmann HE, Agudo A, et al Secondhand smoke exposure in adulthood and risk of lung cancer among never smokers: a pooled analysis of two large studies. Int J Cancer 2004; 109(1):125–31. doi: 10.1002/ijc.11682 14735478 10.1002/ijc.11682

[34] Raaschou-Nielsen O, Andersen ZJ, Beelen R, Samoli E, Stafoggia M, Weinmayr G, et al Air pollution and lung cancer incidence in 17 European cohorts: prospective analyses from the European Study of Cohorts for Air Pollution Effects (ESCAPE). Lancet Oncol 2013; 14(9):813–22. doi: 10.1016/S1470-2045(13)70279-1 23849838 10.1016/S1470-2045(13)70279-1

[35] Richiardi L, Bellocco R, Zugna D. Mediation analysis in epidemiology: methods, interpretation and bias. Int J Epidemiol 2013; 42(5):1511–9. doi: 10.1093/ije/dyt127 24019424 10.1093/ije/dyt127

[36] Rushton L, Hutchings S, Brown T. The burden of cancer at work: estimation as the first step to prevention. Occup Environ Med 2008; 65(12):789–800. doi: 10.1136/oem.2007.037002 18079154 10.1136/oem.2007.037002

[37] Menvielle G, Boshuizen H, Kunst AE, Vineis P, Dalton SO, Bergmann MM, et al Occupational exposures contribute to educational inequalities in lung cancer incidence among men: Evidence from the EPIC prospective cohort study. Int J Cancer 2010; 126(8):1928–35. doi: 10.1002/ijc.24924 19810107 10.1002/ijc.24924 PMC2873305

[38] Behrens T, Groß I, Siemiatycki J, Conway DI, Olsson AC, Stücker I, et al Occupational prestige, social mobility in men and the association with lung cancer. BMC Cancer 2016; 16:395 doi: 10.1186/s12885-016-2432-9 27388894 10.1186/s12885-016-2432-9 PMC4936282

[39] Wadsworth ME. Health inequalities in the life course perspective. Soc Sci Med 1997; 44(6):859–69. 9080567 10.1016/s0277-9536(96)00187-6

[40] Hystad P, Carpiano RM, Demers PA, Johnson KC, Brauer M. Neighbourhood socioeconomic status and individual lung cancer risk: evaluating long-term exposure measures and mediating mechanisms. Soc Sci Med 2013; 97:95–103. doi: 10.1016/j.socscimed.2013.08.005 24161094 10.1016/j.socscimed.2013.08.005

[41] Louwman WJ, van Lenthe FJ, Coebergh JW, Mackenbach JP. Behaviour partly explains educational differences in cancer incidence in the south-eastern Netherlands: the longitudinal GLOBE study. Eur J Cancer Prev 2004; 13(2):119–25. 15100578 10.1097/00008469-200404000-00005

[42] Galobardes B. Shaw M, Lawlor DA, Lynch JW, Davey Smith G. Indicators of socioeconomic position (part 1). J Epidemiol Community Health 2006; 60(1):7–12. doi: 10.1136/jech.2004.023531 16361448 10.1136/jech.2004.023531 PMC2465546

[43] Pukkala E, Martinsen JI, Lynge E, Gunnarsdottir HK, Sparén P, Tryggvadottir L, et al Occupation and cancer—follow-up of 15 million people in five Nordic countries. Acta Oncol 2009; 48(5):646–790. doi: 10.1080/02841860902913546 19925375 10.1080/02841860902913546

[44] Veglia F, Vineis P, Overvad K, Boeing H, Bergmann M, Trichopoulou A, et al Occupational exposures, environmental tobacco smoke, and lung cancer. Epidemiology 2007; 18(6):769–75. 18062064 10.1097/ede.0b013e318142c8a1

[45] Robertson T, Batty GD, Der G, Green MJ, McGlynn LM, McIntyre A, et al Is telomere length socially patterned? Evidence from the West of Scotland Twenty-07 Study. PLoS ONE 2012; 7(7):e41805 doi: 10.1371/journal.pone.0041805 22844525 10.1371/journal.pone.0041805 PMC3402400

