# Supplementary material for: Lung cancer and socioeconomic status in a pooled analysis of case-control studies
Source: PLoS One. 2018 Feb 20;13(2):e0192999. doi: 10.1371/journal.pone.0192999 (PMC5819792; doi:10.1371/journal.pone.0192999)
Supplement: S1 Table — (DOCX) [file pone.0192999.s001.docx]

| **S1 Table.** Estimated lung cancer risks (OR) with 95% confidence intervals (CI) for ISEI categories based on quarters of the score range. | | | | | | |
| --- | --- | --- | --- | --- | --- | --- |
| SES indicator – gender | Cases | | Controls | | Model 1^a^ OR (95%-CI) | Model 2^b^ OR (95%-CI) |
|  | n | % | n | % |  |  |
| Longest job – men |  |  |  |  |  |  |
| ^1^st quarter (71-90) | 591 | 4.3 | 1482 | 9.0 | 1.00 | 1.00 |
| ^2^nd quarter (51-70) | 2449 | 17.8 | 4297 | 26.1 | 1.42 (1.28-1.59) | 1.18 (1.04-1.34) |
| ^3^rd quarter (30-50) | 8415 | 61.1 | 8471 | 51.4 | 2.49 (2.25-2.76) | 1.80 (1.60-2.02) |
| ^4^th quarter (10-29) | 2317 | 16.8 | 2230 | 13.5 | 2.59 (2.31-2.90) | 1.84 (1.61-2.09) |
| *Test for trend* |  |  |  |  | *P < 0.001* | *P < 0.001* |
| Longest job – women |  |  |  |  |  |  |
| ^1^st quarter (71-90) | 146 | 4.5 | 293 | 6.7 | 1.00 | 1.00 |
| ^2^nd quarter (51-70) | 1002 | 30.8 | 1534 | 34.8 | 1.27 (1.02-1.58) | 1.16 (0.91-1.48) |
| ^3^rd quarter (30-50) | 1218 | 37.5 | 1600 | 36.3 | 1.44 (1.16-1.79) | 1.28 (1.00-1.63) |
| ^4^th quarter (10-29) | 883 | 27.2 | 978 | 22.2 | 1.72 (1.37-2.15) | 1.54 (1.20-1.98) |
| *Test for trend* |  |  |  |  | *P < 0.001* | *P < 0.001* |
| First job – men |  |  |  |  |  |  |
| ^1^st quarter (71-90) | 402 | 2.9 | 930 | 5.6 | 1.00 | 1.00 |
| ^2^nd quarter (51-70) | 1401 | 10.2 | 2633 | 16.0 | 1.21 (1.06-1.39) | 0.99 (0.85-1.16) |
| ^3^rd quarter (30-50) | 8906 | 64.7 | 9631 | 58.4 | 2.05 (1.82-2.32) | 1.44 (1.26-1.66) |
| ^4^th quarter (10-29) | 3063 | 22.2 | 3286 | 19.9 | 2.00 (1.76-2.28) | 1.43 (1.24-1.66) |
| *Test for trend* |  |  |  |  | *P < 0.001* | *P < 0.001* |
| First job – women |  |  |  |  |  |  |
| ^1^st quarter (71-90) | 124 | 3.8 | 214 | 4.9 | 1.00 | 1.00 |
| ^2^nd quarter (51-70) | 827 | 25.5 | 1331 | 30.2 | 1.04 (0.82-1.32) | 1.13 (0.86-1.48) |
| ^3^rd quarter (30-50) | 1343 | 41.3 | 1704 | 38.7 | 1.29 (1.01-1.63) | 1.31 (1.00-1.72) |
| ^4^th quarter (10-29) | 955 | 29.4 | 1156 | 26.2 | 1.29 (1.01-1.65) | 1.34 (1.02-1.77) |
| *Test for trend* |  |  |  |  | *P < 0.001* | *P = 0.003* |
| Last job – men |  |  |  |  |  |  |
| ^1^st quarter (71-90) | 558 | 4.1 | 1447 | 8.8 | 1.00 | 1.00 |
| ^2^nd quarter (51-70) | 2676 | 19.4 | 4667 | 28.3 | 1.47 (1.31-1.64) | 1.21 (1.07-1.37) |
| ^3^rd quarter (30-50) | 8084 | 58.7 | 8047 | 48.8 | 2.59 (2.33-2.87) | 1.85 (1.64-2.08) |
| ^4^th quarter (10-29) | 2454 | 17.8 | 2319 | 14.1 | 2.69 (2.40-3.02) | 1.88 (1.65-2.14) |
| *Test for trend* |  |  |  |  | *P < 0.001* | *P < 0.001* |
| Last job – women |  |  |  |  |  |  |
| ^1^st quarter (71-90) | 141 | 4.3 | 283 | 6.4 | 1.00 | 1.00 |
| ^2^nd quarter (51-70) | 993 | 30.6 | 1562 | 35.5 | 1.23 (0.99-1.54) | 1.07 (0.84-1.37) |
| ^3^rd quarter (30-50) | 1182 | 36.4 | 1565 | 35.5 | 1.42 (1.14-1.77) | 1.23 (0.96-1.57) |
| ^4^th quarter (10-29) | 933 | 28.7 | 995 | 22.6 | 1.76 (1.41-2.21) | 1.50 (1.16-1.93) |
| *Test for trend* |  |  |  |  | *P < 0.001* | *P < 0.001* |
| Highest ISEI – men |  |  |  |  |  |  |
| ^1^st quarter (71-90) | 1005 | 7.3 | 2254 | 13.7 | 1.00 | 1.00 |
| ^2^nd quarter (51-70) | 3375 | 24.5 | 5291 | 32.1 | 1.44 (1.32-1.58) | 1.24 (1.12-1.36) |
| ^3^rd quarter (30-50) | 8794 | 63.9 | 8262 | 50.1 | 2.41 (2.22-2.61) | 1.84 (1.68-2.02) |
| ^4^th quarter (10-29) | 598 | 4.3 | 673 | 4.1 | 2.02 (1.77-2.31) | 1.61 (1.38-1.88) |
| *Test for trend* |  |  |  |  | *P < 0.001* | *P < 0.001* |
| Highest ISEI – women |  |  |  |  |  |  |
| ^1^st quarter (71-90) | 210 | 6.5 | 417 | 9.5 | 1.00 | 1.00 |
| ^2^nd quarter (51-70) | 1273 | 39.2 | 1923 | 43.7 | 1.26 (1.05-1.52) | 1.17 (0.95-1.44) |
| ^3^rd quarter (30-50) | 1369 | 42.1 | 1637 | 37.2 | 1.56 (1.29-1.87) | 1.47 (1.19-1.81) |
| ^4^th quarter (10-29) | 397 | 12.2 | 428 | 9.7 | 1.70 (1.36-2.12) | 1.74 (1.36-2.24) |
| *Test for trend* |  |  |  |  | *P < 0.001* | *P < 0.001* |
| Lowest ISEI – men |  |  |  |  |  |  |
| ^1^st quarter (71-90) | 218 | 1.6 | 590 | 3.6 | 1.00 | 1.00 |
| ^2^nd quarter (51-70) | 992 | 7.2 | 2053 | 12.5 | 1.27 (1.07-1.52) | 1.00 (0.82-1.22) |
| ^3^rd quarter (30-50) | 6429 | 46.7 | 7701 | 46.7 | 2.16 (1.84-2.53) | 1.42 (1.18-1.71) |
| ^4^th quarter (10-29) | 6133 | 44.5 | 6136 | 37.2 | 2.51 (2.13-2.95) | 1.58 (1.31-1.90) |
| *Test for trend* |  |  |  |  | *P < 0.001* | *P < 0.001* |
| Lowest ISEI – women |  |  |  |  |  |  |
| ^1^st quarter (71-90) | 83 | 2.6 | 139 | 3.2 | 1.00 | 1.00 |
| ^2^nd quarter (51-70) | 587 | 18.1 | 964 | 21.9 | 1.00 (0.74-1.34) | 1.00 (0.72-1.39) |
| ^3^rd quarter (30-50) | 1034 | 31.8 | 1474 | 33.5 | 1.13 (0.85-1.50) | 1.08 (0.78-1.50) |
| ^4^th quarter (10-29) | 1545 | 47.6 | 1828 | 41.5 | 1.34 (1.01-1.78) | 1.19 (0.86-1.65) |
| *Test for trend* |  |  |  |  | *P < 0.001* | *P = 0.012* |
| ^a^ Adjusted for log(age) and study center  ^b^ Adjusted for log(age), study center, smoking status incl. time since quitting (current smoker, quitted 2-5, 6-10, 11-15, 16-25, 26-35 or >35 years before interview/diagnosis, only other types of tobacco, non-smoker) and cigarette pack-years (log(py+1)) | | | | | | |
