# Supplementary material for: Lung cancer and socioeconomic status in a pooled analysis of case-control studies
Source: PLoS One. 2018 Feb 20;13(2):e0192999. doi: 10.1371/journal.pone.0192999 (PMC5819792; doi:10.1371/journal.pone.0192999)
Supplement: S2 Table — (DOCX) [file pone.0192999.s002.docx]

| **S2 Table.** Estimated lung cancer risks (OR) with 95% confidence intervals (CI) for ESeC categories. | | | | | | |
| --- | --- | --- | --- | --- | --- | --- |
| SES indicator – gender | Cases | | Controls | | Model 1^a^ OR (95%-CI) | Model 2^b^ OR (95%-CI) |
|  | n | % | n | % |  |  |
| Longest job – men |  |  |  |  |  |  |
| The Salariat | 3262 | 23.7 | 5517 | 33.5 | 1.00 | 1.00 |
| Intermediate | 1888 | 13.7 | 2819 | 17.1 | 1.10 (1.02-1.18) | 1.08 (0.99-1.17) |
| Working Class | 8622 | 62.6 | 8144 | 49.4 | 1.79 (1.70-1.89) | 1.53 (1.44-1.63) |
| *Test for trend* |  |  |  |  | *P < 0.001* | *P < 0.001* |
| Longest job – women |  |  |  |  |  |  |
| The Salariat | 830 | 25.5 | 1405 | 31.9 | 1.00 | 1.00 |
| Intermediate | 684 | 21.1 | 950 | 21.6 | 1.22 (1.07-1.40) | 1.22 (1.05-1.42) |
| Working Class | 1735 | 53.4 | 2050 | 46.5 | 1.41 (1.27-1.58) | 1.34 (1.19-1.52) |
| *Test for trend* |  |  |  |  | *P < 0.001* | *P < 0.001* |
| First job – men |  |  |  |  |  |  |
| The Salariat | 1835 | 13.3 | 3270 | 19.8 | 1.00 | 1.00 |
| Intermediate | 1467 | 10.7 | 2094 | 12.7 | 1.19 (1.09-1.30) | 1.13 (1.02-1.25) |
| Working Class | 10470 | 76.0 | 11116 | 67.5 | 1.60 (1.50-1.71) | 1.35 (1.25-1.45) |
| *Test for trend* |  |  |  |  | *P < 0.001* | *P < 0.001* |
| First job – women |  |  |  |  |  |  |
| The Salariat | 700 | 21.5 | 1119 | 25.4 | 1.00 | 1.00 |
| Intermediate | 620 | 19.1 | 903 | 20.5 | 1.13 (0.98-1.30) | 1.10 (0.93-1.29) |
| Working Class | 1929 | 59.4 | 2383 | 54.1 | 1.27 (1.13-1.43) | 1.19 (1.04-1.35) |
| *Test for trend* |  |  |  |  | *P < 0.001* | *P = 0.008* |
| Last job – men |  |  |  |  |  |  |
| The Salariat | 3315 | 24.1 | 5722 | 34.7 | 1.00 | 1.00 |
| Intermediate | 1957 | 14.2 | 2886 | 17.5 | 1.15 (1.07-1.23) | 1.09 (1.01-1.19) |
| Working Class | 8500 | 61.7 | 7872 | 47.8 | 1.86 (1.77-1.97) | 1.57 (1.48-1.67) |
| *Test for trend* |  |  |  |  | *P < 0.001* | *P < 0.001* |
| Last job – women |  |  |  |  |  |  |
| The Salariat | 837 | 25.8 | 1457 | 33.1 | 1.00 | 1.00 |
| Intermediate | 667 | 20.5 | 922 | 20.9 | 1.27 (1.11-1.45) | 1.26 (1.09-1.47) |
| Working Class | 1745 | 53.7 | 2026 | 46.0 | 1.47 (1.32-1.64) | 1.40 (1.23-1.58) |
| *Test for trend* |  |  |  |  | *P < 0.001* | *P < 0.001* |
| ^a^ Adjusted for log(age) and study center  ^b^ Adjusted for log(age), study center, smoking status incl. time since quitting (current smoker, quitted 2-5, 6-10, 11-15, 16-25, 26-35 or >35 years before interview/diagnosis, only other types of tobacco, non-smoker) and cigarette pack-years (log(py+1)) | | | | | | |
