# Supplementary material for: Lung cancer and socioeconomic status in a pooled analysis of case-control studies
Source: PLoS One. 2018 Feb 20;13(2):e0192999. doi: 10.1371/journal.pone.0192999 (PMC5819792; doi:10.1371/journal.pone.0192999)
Supplement: S3 Table — (DOCX) [file pone.0192999.s003.docx]

| **S3 Table.** Estimated lung cancer risks (OR) with 95% confidence intervals (CI) for categories of sums of unemployed years. | | | | | | |
| --- | --- | --- | --- | --- | --- | --- |
| Years of unemployment | Cases | | Controls | | Model 1^a^ OR (95%-CI) | Model 2^b^ OR (95%-CI) |
|  | n | % | n | % |  |  |
| Men |  |  |  |  |  |  |
| Never unemployed | 12125 | 88.0 | 14885 | 90.3 | 1.00 | 1.00 |
| <= 1 year | 518 | 3.8 | 576 | 3.5 | 1.06 (0.93-1.20) | 1.00 (0.87-1.15) |
| > 1 - 5 years | 696 | 5.1 | 689 | 4.2 | 1.19 (1.06-1.33) | 1.02 (0.90-1.15) |
| > 5 - 10 years | 261 | 1.9 | 180 | 1.1 | 1.78 (1.46-2.16) | 1.43 (1.15-1.79) |
| > 10 years | 172 | 1.2 | 150 | 0.9 | 1.55 (1.24-1.94) | 1.15 (0.89-1.48) |
| Women |  |  |  |  |  |  |
| Never unemployed | 2879 | 88.6 | 3955 | 89.8 | 1.00 | 1.00 |
| <= 1 year | 106 | 3.3 | 127 | 2.9 | 1.04 (0.80-1.36) | 0.85 (0.62-1.15) |
| > 1 - 5 years | 154 | 4.7 | 180 | 4.1 | 1.08 (0.87-1.36) | 0.98 (0.76-1.26) |
| > 5 - 10 years | 47 | 1.4 | 72 | 1.6 | 0.87 (0.59-1.26) | 0.83 (0.54-1.27) |
| > 10 years | 63 | 1.9 | 71 | 1.6 | 1.25 (0.88-1.78) | 0.92 (0.63-1.36) |
| ^a^ Adjusted for log(age) and study center  ^b^ Adjusted for log(age), study center, smoking status incl. time since quitting (current smoker, quitted 2-5, 6-10, 11-15, 16-25, 26-35 or >35 years before interview/diagnosis, only other types of tobacco, non-smoker) and cigarette pack-years (log(py+1)) | | | | | | |
