# Supplementary material for: Lung cancer and socioeconomic status in a pooled analysis of case-control studies
Source: PLoS One. 2018 Feb 20;13(2):e0192999. doi: 10.1371/journal.pone.0192999 (PMC5819792; doi:10.1371/journal.pone.0192999)
Supplement: S4 Table — (DOCX) [file pone.0192999.s004.docx]

| **S4 Table.** Estimated lung cancer risks (OR) with 95% confidence intervals (CI) for ISEI categories based on gender-specific quartiles of the distribution of the controls. | | | | | | |
| --- | --- | --- | --- | --- | --- | --- |
| SES indicator – gender | Cases | | Controls | | Model 1^a^ OR (95%-CI) | Model 2^b^ OR (95%-CI) |
|  | n | % | n | % |  |  |
| Longest job – men |  |  |  |  |  |  |
| 1^st^ quartile (59-90) | 1749 | 12.7 | 3719 | 22.6 | 1.00 | 1.00 |
| 2^nd^ quartile (42-58) | 2952 | 21.4 | 4371 | 26.5 | 1.44 (1.34-1.55) | 1.31 (1.20-1.43) |
| 3^rd^ quartile (34-41) | 3989 | 29.0 | 3910 | 23.7 | 2.25 (2.09-2.42) | 1.73 (1.60-1.88) |
| 4^th^ quartile (10-33) | 5082 | 36.9 | 4480 | 27.2 | 2.42 (2.25-2.59) | 1.88 (1.73-2.03) |
| *Test for trend* |  |  |  |  | *P < 0.001* | *P < 0.001* |
| Longest job – women |  |  |  |  |  |  |
| 1^st^ quartile (59-90) | 438 | 13.5 | 793 | 18.0 | 1.00 | 1.00 |
| 2^nd^ quartile (47-58) | 844 | 26.0 | 1249 | 28.4 | 1.25 (1.08-1.45) | 1.22 (1.03-1.44) |
| 3^rd^ quartile (33-46) | 948 | 29.2 | 1237 | 28.1 | 1.39 (1.20-1.61) | 1.30 (1.10-1.54) |
| 4^th^ quartile (10-32) | 1019 | 31.4 | 1126 | 25.6 | 1.63 (1.41-1.89) | 1.55 (1.31-1.83) |
| *Test for trend* |  |  |  |  | *P < 0.001* | *P < 0.001* |
| First job – men |  |  |  |  |  |  |
| 1^st^ quartile (49-90) | 2219 | 16.1 | 4011 | 24.3 | 1.00 | 1.00 |
| 2^nd^ quartile (37-48) | 3182 | 23.1 | 4068 | 24.7 | 1.44 (1.34-1.55) | 1.21 (1.12-1.32) |
| 3^rd^ quartile (32-36) | 4321 | 31.4 | 4183 | 25.4 | 1.84 (1.72-1.97) | 1.44 (1.34-1.56) |
| 4^th^ quartile (10-31) | 4050 | 29.4 | 4218 | 25.6 | 1.69 (1.58-1.81) | 1.40 (1.29-1.51) |
| *Test for trend* |  |  |  |  | *P < 0.001* | *P < 0.001* |
| First job – women |  |  |  |  |  |  |
| 1^st^ quartile (59-90) | 818 | 25.2 | 1311 | 29.8 | 1.00 | 1.00 |
| 2^nd^ quartile (43-58) | 555 | 17.1 | 878 | 19.9 | 1.03 (0.89-1.18) | 1.02 (0.87-1.19) |
| 3^rd^ quartile (29-42) | 959 | 29.5 | 1098 | 24.9 | 1.35 (1.19-1.53) | 1.24 (1.08-1.43) |
| 4^th^ quartile (10-28) | 917 | 28.2 | 1118 | 25.4 | 1.23 (1.08-1.39) | 1.17 (1.02-1.36) |
| *Test for trend* |  |  |  |  | *P < 0.001* | *P = 0.005* |
| Last job – men |  |  |  |  |  |  |
| 1^st^ quartile (59-90) | 1859 | 13.5 | 3969 | 24.1 | 1.00 | 1.00 |
| 2^nd^ quartile (44-58) | 2754 | 20.0 | 4065 | 24.7 | 1.46 (1.35-1.57) | 1.31 (1.20-1.42) |
| 3^rd^ quartile (34-43) | 4180 | 30.4 | 4123 | 25.0 | 2.23 (2.08-2.40) | 1.74 (1.60-1.89) |
| 4^th^ quartile (10-33) | 4979 | 36.2 | 4323 | 26.2 | 2.45 (2.29-2.63) | 1.89 (1.75-2.05) |
| *Test for trend* |  |  |  |  | *P < 0.001* | *P < 0.001* |
| Last job – women |  |  |  |  |  |  |
| 1^st^ quartile (59-90) | 451 | 13.9 | 840 | 19.1 | 1.00 | 1.00 |
| 2^nd^ quartile (47-58) | 813 | 25.0 | 1219 | 27.7 | 1.26 (1.09-1.47) | 1.21 (1.03-1.43) |
| 3^rd^ quartile (33-46) | 913 | 28.1 | 1217 | 27.6 | 1.39 (1.20-1.61) | 1.32 (1.12-1.55) |
| 4^th^ quartile (10-32) | 1072 | 33.0 | 1129 | 25.6 | 1.75 (1.51-2.02) | 1.61 (1.37-1.90) |
| *Test for trend* |  |  |  |  | *P < 0.001* | *P < 0.001* |
| Highest ISEI – men |  |  |  |  |  |  |
| 1^st^ quartile (65-90) | 1975 | 14.3 | 3999 | 24.3 | 1.00 | 1.00 |
| 2^nd^ quartile (49-64) | 2997 | 21.8 | 4222 | 25.6 | 1.40 (1.30-1.50) | 1.26 (1.16-1.37) |
| 3^rd^ quartile (38-48) | 3806 | 27.6 | 4082 | 24.8 | 1.93 (1.80-2.07) | 1.56 (1.44-1.69) |
| 4^th^ quartile (10-37) | 4994 | 36.3 | 4177 | 25.3 | 2.42 (2.26-2.59) | 1.89 (1.74-2.04) |
| *Test for trend* |  |  |  |  | *P < 0.001* | *P < 0.001* |
| Highest ISEI – women |  |  |  |  |  |  |
| 1^st^ quartile (60-90) | 538 | 16.6 | 954 | 21.7 | 1.00 | 1.00 |
| 2^nd^ quartile (53-59) | 817 | 25.1 | 1203 | 27.3 | 1.16 (1.01-1.34) | 1.09 (0.93-1.28) |
| 3^rd^ quartile (42-52) | 695 | 21.4 | 985 | 22.4 | 1.22 (1.05-1.41) | 1.25 (1.06-1.48) |
| 4^th^ quartile (10-41) | 1199 | 36.9 | 1263 | 28.7 | 1.58 (1.38-1.81) | 1.54 (1.32-1.80) |
| *Test for trend* |  |  |  |  | *P < 0.001* | *P < 0.001* |
| Lowest ISEI – men |  |  |  |  |  |  |
| 1^st^ quartile (42-90) | 2011 | 14.6 | 3954 | 24.0 | 1.00 | 1.00 |
| 2^nd^ quartile (33-41) | 3323 | 24.1 | 4245 | 25.8 | 1.54 (1.43-1.65) | 1.27 (1.17-1.38) |
| 3^rd^ quartile (27-32) | 3416 | 24.8 | 3381 | 20.5 | 1.90 (1.77-2.05) | 1.51 (1.39-1.64) |
| 4^th^ quartile (10-26) | 5022 | 36.5 | 4900 | 29.7 | 1.93 (1.81-2.07) | 1.54 (1.42-1.66) |
| *Test for trend* |  |  |  |  | *P < 0.001* | *P < 0.001* |
| Lowest ISEI – women |  |  |  |  |  |  |
| 1^st^ quartile (52-90) | 641 | 19.7 | 1036 | 23.5 | 1.00 | 1.00 |
| 2^nd^ quartile (34-51) | 747 | 23.0 | 1136 | 25.8 | 1.06 (0.92-1.21) | 1.03 (0.88-1.2) |
| 3^rd^ quartile (25-33) | 1018 | 31.3 | 1204 | 27.3 | 1.33 (1.17-1.52) | 1.13 (0.98-1.32) |
| 4^th^ quartile (10-24) | 843 | 25.9 | 1029 | 23.4 | 1.26 (1.10-1.45) | 1.18 (1.01-1.38) |
| *Test for trend* |  |  |  |  | *P < 0.001* | *P = 0.019* |
| ^a^ Adjusted for log(age) and study center  ^b^ Adjusted for log(age), study center, smoking status incl. time since quitting (current smoker, quitted 2-5, 6-10, 11-15, 16-25, 26-35 or >35 years before interview/diagnosis, only other types of tobacco, non-smoker) and cigarette pack-years (log(py+1)) | | | | | | |
