# Supplementary material for: Lung cancer and socioeconomic status in a pooled analysis of case-control studies
Source: PLoS One. 2018 Feb 20;13(2):e0192999. doi: 10.1371/journal.pone.0192999 (PMC5819792; doi:10.1371/journal.pone.0192999)
Supplement: S5 Table — Contains the following: Table A. Correlation of ISEI job periods by Spearman's rank correlation coefficient. Table B. Correlation of ESeC job periods. (DOCX) [file pone.0192999.s005.docx]

**S5 Tables.** Correlation of job periods.

| **Table S5A.** Correlation of ISEI job periods by Spearman's rank correlation coefficient | | | | | |
| --- | --- | --- | --- | --- | --- |
| ISEI job period (quarters of ISEI range) | Longest | First | Last | Highest | Lowest |
| Longest | 1.000 |  |  |  |  |
| First | .552 | 1.000 |  |  |  |
| Last | .849 | .494 | 1.000 |  |  |
| Highest | .772 | .569 | .785 | 1.000 |  |
| Lowest | .614 | .747 | .612 | .466 | 1.000 |
| ISEI job period (quartiles by control distribution of men) |  | S |  |  |  |
| Longest | 1.000 |  |  |  |  |
| First | .486 | 1.000 |  |  |  |
| Last | .851 | .437 | 1.000 |  |  |
| Highest | .740 | .522 | .755 | 1.000 |  |
| Lowest | .543 | .688 | .540 | .414 | 1.000 |
| ISEI job period (quartiles by control distribution of women) |  |  |  |  |  |
| Longest | 1.000 |  |  |  |  |
| First | .607 | 1.000 |  |  |  |
| Last | .872 | .563 | 1.000 |  |  |
| Highest | .766 | .663 | .763 | 1.000 |  |
| Lowest | .678 | .757 | .677 | .574 | 1.000 |

| **Table S5B.** Correlation of ESeC job periods. |  |
| --- | --- |
| ESeC – job periods | Cramérs V |
| Longest – first | .474 |
| Longest – last | .803 |
| First – last | .401 |
