# Supplementary material for: Lung cancer and socioeconomic status in a pooled analysis of case-control studies
Source: PLoS One. 2018 Feb 20;13(2):e0192999. doi: 10.1371/journal.pone.0192999 (PMC5819792; doi:10.1371/journal.pone.0192999)
Supplement: S6 Table — (DOCX) [file pone.0192999.s006.docx]

| **S6 Table.** Estimated lung cancer risks (OR) with 95% confidence intervals (CI) for occupational SES by gender – with additional adjustment for education or ‘list A’. | | | | | | |
| --- | --- | --- | --- | --- | --- | --- |
| SES indicator – gender | Cases | | Controls | | Model 3^1)^ OR (95%-CI) | Model 4^2)^ OR (95%-CI) |
|  | n | % | n | % |  |  |
| ISEI^3)^ longest job – men |  |  |  |  |  |  |
| 1^st^ quarter (71-90) | 591 | 4.3 | 1482 | 9.0 | 1.00 | 1.00 |
| 2^nd^ quarter (51-70) | 2449 | 17.8 | 4297 | 26.1 | 1.09 (0.95-1.23) | 1.18 (1.04-1.33) |
| 3^rd^ quarter (30-50) | 8415 | 61.1 | 8471 | 51.4 | 1.46 (1.29-1.66) | 1.74 (1.55-1.95) |
| 4^th^ quarter (10-29) | 2317 | 16.8 | 2230 | 13.5 | 1.44 (1.25-1.66) | 1.77 (1.56-2.02) |
| *Test for trend* |  |  |  |  | *P < 0.001* | *P < 0.001* |
| ISEI^c)^ longest job – women |  |  |  |  |  |  |
| 1^st^ quarter (71-90) | 146 | 4.5 | 293 | 6.7 | 1.00 | 1.00 |
| 2^nd^ quarter (51-70) | 1002 | 30.8 | 1534 | 34.8 | 1.02 (0.79-1.32) | 1.16 (0.91-1.48) |
| 3^rd^ quarter (30-50) | 1218 | 37.5 | 1600 | 36.3 | 1.02 (0.78-1.33) | 1.27 (0.99-1.62) |
| 4^th^ quarter (10-29) | 883 | 27.2 | 978 | 22.2 | 1.17 (0.88-1.55) | 1.52 (1.18-1.95) |
| *Test for trend* |  |  |  |  | *P = 0.125* | *P < 0.001* |
| ESeC longest job – men |  |  |  |  |  |  |
| The Salariat | 3262 | 23.7 | 5517 | 33.5 | 1.00 | 1.00 |
| Intermediate | 1888 | 13.7 | 2819 | 17.1 | 1.01 (0.93-1.11) | 1.07 (0.99-1.17) |
| Working Class | 8622 | 62.6 | 8144 | 49.4 | 1.33 (1.24-1.43) | 1.49 (1.40-1.58) |
| *Test for trend* |  |  |  |  | *P < 0.001* | *P < 0.001* |
| ESeC longest job – women |  |  |  |  |  |  |
| The Salariat | 830 | 25.5 | 1405 | 31.9 | 1.00 | 1.00 |
| Intermediate | 684 | 21.1 | 950 | 21.6 | 1.16 (1.00-1.36) | 1.22 (1.05-1.41) |
| Working Class | 1735 | 53.4 | 2050 | 46.5 | 1.16 (1.01-1.34) | 1.33 (1.17-1.50) |
| *Test for trend* |  |  |  |  | *P = 0.039* | *P < 0.001* |
| ^1)^ Adjusted for log(age), study center, smoking status incl. time since quitting (current smoker, quitted 2-5, 6-10, 11-15, 16-25, 26-35 or >35 years before interview/diagnosis, only other types of tobacco, non-smoker) and cigarette pack-years (log(py+1)) and education  ^2)^ Adjusted for log(age), study center, smoking status incl. time since quitting (current smoker, quitted 2-5, 6-10, 11-15, 16-25, 26-35 or >35 years before interview/diagnosis, only other types of tobacco, non-smoker) and cigarette pack-years (log(py+1)) and education  ^3)^ Categories by quarters of ISEI range | | | | | | |
