# Supplementary material for: Lung cancer and socioeconomic status in a pooled analysis of case-control studies
Source: PLoS One. 2018 Feb 20;13(2):e0192999. doi: 10.1371/journal.pone.0192999 (PMC5819792; doi:10.1371/journal.pone.0192999)
Supplement: S7 Table — (DOCX) [file pone.0192999.s007.docx]

| **S7 Table.** Association of SES (ISEI – longest job) and lung cancer in participants with last residence in an urban area and age < 63 years. | | | | | | |
| --- | --- | --- | --- | --- | --- | --- |
|  | Men | | | Women | | |
| ISEI^a^ | Cases | Controls | OR (95%-CI)^b^ | Cases | Controls | OR (95%-CI)^b^ |
| 1^st^ quarter (71-90) | 121 | 281 | 1.00 | 45 | 78 | 1.00 |
| 2^nd^ quarter (51-70) | 491 | 830 | 1.12 (0.85-1.48) | 285 | 379 | 1.00 (0.63-1.60) |
| 3^rd^ quarter (30-50) | 1625 | 1558 | 1.55 (1.20-2.01) | 317 | 389 | 1.01 (0.63-1.60) |
| 4^th^ quarter (10-29) | 436 | 383 | 1.56 (1.17-2.09) | 184 | 175 | 1.27 (0.77-2.09) |
| *Test for trend* |  |  | *P < 0.001* |  |  | *P = 0.218* |
| ^a^ Categories by quarters of ISEI-range  ^b^ Odds ratio with 95% confidence interval – adjusted for log(age), study center, smoking status incl. time since quitting (current smoker, quitted 2-5, 6-10, 11-15, 16-25, 26-35 or >35 years before interview/diagnosis, only other types of tobacco, non-smoker) and cigarette pack-years (log(py+1)) | | | | | | |
