# Supplementary material for: Lung cancer and socioeconomic status in a pooled analysis of case-control studies
Source: PLoS One. 2018 Feb 20;13(2):e0192999. doi: 10.1371/journal.pone.0192999 (PMC5819792; doi:10.1371/journal.pone.0192999)
Supplement: S8 Table — (DOCX) [file pone.0192999.s008.docx]

| **S8 Table.** Association of SES (ISEI – longest job) and lung cancer by study region. | | | | | | |
| --- | --- | --- | --- | --- | --- | --- |
|  | Men | | | Women | | |
| Study region | Cases | Controls | OR (95%-CI)^a^ | Cases | Controls | OR (95%-CI)^a^ |
| Categories by quarters of ISEI range | | | | | | |
| Northern / Central Europe |  |  |  |  |  |  |
| 1^st^ quarter (71-90) | 320 | 971 | 1.00 | 40 | 90 | 1.00 |
| 2^nd^ quarter (51-70) | 1270 | 2500 | 1.25 (1.06-1.47) | 386 | 602 | 1.69 (1.08-2.65) |
| 3^rd^ quarter (30-50) | 4491 | 4758 | 2.12 (1.82-2.47) | 577 | 668 | 2.22 (1.42-3.47) |
| 4^th^ quarter (10-29) | 1217 | 1187 | 2.25 (1.89-2.67) | 437 | 439 | 2.74 (1.74-4.31) |
| *Test for trend* |  |  | *P < 0.001* |  |  | *P < 0.001* |
| Eastern Europe |  |  |  |  |  |  |
| 1^st^ quarter (71-90) | 106 | 168 | 1.00 | 52 | 50 | 1.00 |
| 2^nd^ quarter (51-70) | 277 | 406 | 0.98 (0.7-1.36) | 168 | 184 | 0.69 (0.42-1.14) |
| 3^rd^ quarter (30-50) | 1301 | 1109 | 1.38 (1.02-1.85) | 228 | 298 | 0.54 (0.33-0.87) |
| 4^th^ quarter (10-29) | 348 | 309 | 1.30 (0.93-1.82) | 112 | 138 | 0.63 (0.37-1.06) |
| *Test for trend* |  |  | *P = 0.005* |  |  | *P = 0.068* |
| Southern Europe |  |  |  |  |  |  |
| 1^st^ quarter (71-90) | 113 | 193 | 1.00 | 21 | 45 | 1.00 |
| 2^nd^ quarter (51-70) | 712 | 1047 | 1.01 (0.76-1.35) | 224 | 287 | 1.79 (0.97-3.32) |
| 3^rd^ quarter (30-50) | 2143 | 2067 | 1.32 (1.00-1.74) | 216 | 328 | 1.82 (0.98-3.38) |
| 4^th^ quarter (10-29) | 568 | 511 | 1.39 (1.03-1.88) | 160 | 200 | 2.03 (1.08-3.83) |
| *Test for trend* |  |  | *P < 0.001* |  |  | *P = 0.111* |
| Canada |  |  |  |  |  |  |
| 1^st^ quarter (71-90) | 52 | 150 | 1.00 | 33 | 108 | 1.00 |
| 2^nd^ quarter (51-70) | 190 | 344 | 1.29 (0.86-1.93) | 224 | 461 | 1.01 (0.63-1.62) |
| 3^rd^ quarter (30-50) | 480 | 537 | 1.71 (1.17-2.50) | 197 | 306 | 1.05 (0.65-1.71) |
| 4^th^ quarter (10-29) | 184 | 223 | 1.43 (0.94-2.18) | 174 | 201 | 1.28 (0.77-2.12) |
| *Test for trend* |  |  | *P = 0.042* |  |  | *P = 0.184* |
| Categories by gender-specific quartiles^b^ | | | | | | |
| Northern / Central Europe |  |  |  |  |  |  |
| 1^st^ quartile | 1002 | 2425 | 1.00 | 180 | 329 | 1.00 |
| 2^nd^ quartile | 1551 | 2484 | 1.40 (1.25-1.56) | 320 | 466 | 1.39 (1.07-1.81) |
| 3^rd^ quartile | 2076 | 2177 | 1.90 (1.70-2.12) | 443 | 509 | 1.66 (1.28-2.14) |
| 4^th^ quartile | 2669 | 2330 | 2.22 (1.99-2.46) | 497 | 495 | 2.02 (1.57-2.61) |
| *Test for trend* |  |  | *P < 0.001* |  |  | *P < 0.001* |
| Eastern Europe |  |  |  |  |  |  |
| 1^st^ quartile | 290 | 423 | 1.00 | 109 | 111 | 1.00 |
| 2^nd^ quartile | 318 | 385 | 1.10 (0.86-1.40) | 127 | 153 | 0.72 (0.48-1.08) |
| 3^rd^ quartile | 654 | 540 | 1.38 (1.12-1.72) | 188 | 234 | 0.68 (0.46-0.99) |
| 4^th^ quartile | 770 | 644 | 1.38 (1.11-1.71) | 136 | 172 | 0.72 (0.48-1.08) |
| *Test for trend* |  |  | *P < 0.001* |  |  | *P = 0.135* |
| Southern Europe |  |  |  |  |  |  |
| 1^st^ quartile | 305 | 524 | 1.00 | 69 | 100 | 1.00 |
| 2^nd^ quartile | 926 | 1225 | 1.21 (1.00-1.47) | 196 | 265 | 1.20 (0.80-1.80) |
| 3^rd^ quartile | 996 | 932 | 1.50 (1.24-1.81) | 165 | 263 | 1.20 (0.80-1.81) |
| 4^th^ quartile | 1309 | 1137 | 1.58 (1.31-1.91) | 191 | 232 | 1.41 (0.93-2.13) |
| *Test for trend* |  |  | *P < 0.001* |  |  | *P = 0.116* |
| Canada |  |  |  |  |  |  |
| 1^st^ quartile | 152 | 347 | 1.00 | 80 | 253 | 1.00 |
| 2^nd^ quartile | 157 | 277 | 1.13 (0.83-1.55) | 201 | 365 | 1.42 (1.00-2.01) |
| 3^rd^ quartile | 263 | 261 | 1.67 (1.24-2.24) | 152 | 231 | 1.36 (0.93-1.99) |
| 4^th^ quartile | 334 | 369 | 1.43 (1.08-1.89) | 195 | 227 | 1.66 (1.14-2.43) |
| *Test for trend* |  |  | *P = 0.004* |  |  | *P = 0.023* |
| ^a^ Odds ratio with 95% confidence interval – adjusted for log(age), study center, smoking status incl. time since quitting (current smoker, quitted 2-5, 6-10, 11-15, 16-25, 26-35 or >35 years before interview/diagnosis, only other types of tobacco, non-smoker) and cigarette pack-years (log(py+1))  ^b^ Men: 1^st^ quartile (59-90), 2^nd^ quartile (42-58), 3^rd^ quartile (34-41), 4^th^ quartile (10-33)  Women: 1^st^ quartile (59-90), 2^nd^ quartile (47-58), 3^rd^ quartile (33-46), 4^th^ quartile (10-31) | | | | | | |
