# Supplementary material for: Lung cancer and socioeconomic status in a pooled analysis of case-control studies
Source: PLoS One. 2018 Feb 20;13(2):e0192999. doi: 10.1371/journal.pone.0192999 (PMC5819792; doi:10.1371/journal.pone.0192999)
Supplement: S9 Table — (DOCX) [file pone.0192999.s009.docx]

| **S9 Table.** Association of SES (ISEI^a^ – longest job) and lung cancer by birth cohort. | | | | | | |
| --- | --- | --- | --- | --- | --- | --- |
|  | Men | | | Women | | |
| Birth cohort | Cases | Controls | OR (95%-CI)^b^ | Cases | Controls | OR (95%-CI)^b^ |
| <1930 |  |  |  |  |  |  |
| 1^st^ quarter (71-90) | 262 | 545 | 1.00 | 24 | 41 | 1.00 |
| 2^nd^ quarter (51-70) | 949 | 1572 | 1.11 (0.92-1.35) | 229 | 316 | 1.01 (0.56-1.82) |
| 3^rd^ quarter (30-50) | 2864 | 3044 | 1.59 (1.33-1.90) | 309 | 406 | 1.03 (0.58-1.84) |
| 4^th^ quarter (10-29) | 739 | 775 | 1.51 (1.23-1.86) | 279 | 238 | 1.73 (0.96-3.11) |
| *Test for trend* |  |  | *P < 0.001* |  |  | *P < 0.001* |
| 1930-1939 |  |  |  |  |  |  |
| 1^st^ quarter (71-90) | 173 | 436 | 1.00 | 52 | 76 | 1.00 |
| 2^nd^ quarter (51-70) | 800 | 1330 | 1.33 (1.06-1.67) | 295 | 454 | 0.92 (0.59-1.44) |
| 3^rd^ quarter (30-50) | 2980 | 2735 | 2.11 (1.70-2.61) | 382 | 489 | 1.22 (0.78-1.90) |
| 4^th^ quarter (10-29) | 844 | 717 | 2.27 (1.79-2.87) | 286 | 367 | 1.23 (0.78-1.93) |
| *Test for trend* |  |  | *P < 0.001* |  |  | *P = 0.030* |
| >1939 |  |  |  |  |  |  |
| 1^st^ quarter (71-90) | 156 | 501 | 1.00 | 70 | 176 | 1.00 |
| 2^nd^ quarter (51-70) | 700 | 1395 | 1.18 (0.94-1.49) | 478 | 764 | 1.41 (0.99-2.00) |
| 3^rd^ quarter (30-50) | 2571 | 2692 | 1.81 (1.46-2.25) | 527 | 705 | 1.41 (1.00-2.00) |
| 4^th^ quarter (10-29) | 734 | 738 | 1.83 (1.44-2.33) | 318 | 373 | 1.57 (1.08-2.27) |
| *Test for trend* |  |  | *P < 0.001* |  |  | *P = 0.062* |
| ^a^ Categories by quarters of ISEI range  ^b^ Odds ratio with 95% confidence interval – adjusted for log(age), study center, smoking status incl. time since quitting (current smoker, quitted 2-5, 6-10, 11-15, 16-25, 26-35 or >35 years before interview/diagnosis, only other types of tobacco, non-smoker) and cigarette pack-years (log(py+1)) | | | | | | |
