# Supplementary material for: Lung cancer and socioeconomic status in a pooled analysis of case-control studies
Source: PLoS One. 2018 Feb 20;13(2):e0192999. doi: 10.1371/journal.pone.0192999 (PMC5819792; doi:10.1371/journal.pone.0192999)
Supplement: S10 Table — (DOCX) [file pone.0192999.s010.docx]

| **S10 Table.** Association of SES (ISEI^a^ – longest job) and lung cancer by education. | | | | | | |
| --- | --- | --- | --- | --- | --- | --- |
|  | Men | | | Women | | |
| Education | Cases | Controls | OR (95%-CI)^b^ | Cases | Controls | OR (95%-CI)^b^ |
| University | | | | | | |
| 1^st^ quarter (71-90) | 344 | 995 | 1.00 | 113 | 238 | 1.00 |
| 2^nd^ quarter (51-70) | 550 | 1240 | 0.98 (0.81-1.18) | 227 | 435 | 1.19 (0.85-1.66) |
| 3^rd^ quarter (30-50) | 434 | 594 | 1.29 (1.04-1.61) | 130 | 201 | 1.06 (0.72-1.56) |
| 4^th^ quarter (10-29) | 73 | 91 | 1.45 (0.98-2.16) | 18 | 39 | 0.89 (0.43-1.84) |
| *Test for trend* |  |  | *P = 0.009* |  |  | *P = 0.952* |
| Secondary/some college (10-13 years) | | | | | | |
| 1^st^ quarter (71-90) | 118 | 254 | 1.00 | 21 | 43 | 1.00 |
| 2^nd^ quarter (51-70) | 872 | 1587 | 0.98 (0.75-1.29) | 409 | 621 | 1.05 (0.57-1.91) |
| 3^rd^ quarter (30-50) | 1322 | 1899 | 1.18 (0.90-1.53) | 211 | 346 | 0.93 (0.50-1.73) |
| 4^th^ quarter (10-29) | 256 | 355 | 1.12 (0.82-1.53) | 64 | 107 | 0.95 (0.48-1.88) |
| *Test for trend* |  |  | *P = 0.037* |  |  | *P = 0.458* |
| Primary/some secondary (6-9 years) | | | | | | |
| 1^st^ quarter (71-90) | 100 | 177 | 1.00 | 11 | 7 | 1.00 |
| 2^nd^ quarter (51-70) | 790 | 1206 | 1.22 (0.90-1.64) | 289 | 372 | 0.37 (0.12-1.11) |
| 3^rd^ quarter (30-50) | 4511 | 4320 | 1.71 (1.29-2.27) | 625 | 710 | 0.41 (0.14-1.23) |
| 4^th^ quarter (10-29) | 1199 | 1158 | 1.72 (1.28-2.31) | 492 | 488 | 0.48 (0.16-1.42) |
| *Test for trend* |  |  | *P < 0.001* |  |  | *P = 0.094* |
| No formal education/some primary (< 6 years) | | | | | | |
| 1^st^ quarter (71-90) | 21 | 35 | 1.00 | 0 | 3 |  |
| 2^nd^ quarter (51-70) | 176 | 201 | 1.34 (0.69-2.59) | 57 | 92 |  |
| 3^rd^ quarter (30-50) | 1863 | 1518 | 1.77 (0.95-3.30) | 223 | 310 |  |
| 4^th^ quarter (10-29) | 676 | 572 | 1.69 (0.90-3.16) | 280 | 324 |  |
| *Test for trend* |  |  | *P = 0.168* |  |  | *P = 0.033* |
| ^a^ Categories by quarters of ISEI range  ^b^ Odds ratio with 95% confidence interval – adjusted for log(age), study center, smoking status incl. time since quitting (current smoker, quitted 2-5, 6-10, 11-15, 16-25, 26-35 or >35 years before interview/diagnosis, only other types of tobacco, non-smoker) and cigarette pack-years (log(py+1)) | | | | | | |
