# Supplementary material for: Lung cancer and socioeconomic status in a pooled analysis of case-control studies
Source: PLoS One. 2018 Feb 20;13(2):e0192999. doi: 10.1371/journal.pone.0192999 (PMC5819792; doi:10.1371/journal.pone.0192999)
Supplement: S11 Table — (DOCX) [file pone.0192999.s011.docx]

| **S11 Table.** Estimated lung cancer risks (OR) with 95% confidence intervals (CI) for unemployment of more than 1 year. | | | | |
| --- | --- | --- | --- | --- |
| Gender  Ever unemployed > 1 year | Cases | Controls | Model 1^a^ OR (95%-CI) | Model 2^b^ OR (95%-CI) |
| Men |  |  |  |  |
| No | 12682 | 15503 | 1.00 | 1.00 |
| Yes | 1090 | 977 | 1.34 (1.22-1.47) | 1.11 (1.00-1.23) |
| Women |  |  |  |  |
| No | 2996 | 4088 | 1.00 | 1.00 |
| Yes | 253 | 317 | 1.04 (0.87-1.24) | 0.92 (0.75-1.12) |
| ^a^ Adjusted for log(age), study center  ^b^ Adjusted for log(age), study center, smoking status incl. time since quitting (current smoker, quitted 2-5, 6-10, 11-15, 16-25, 26-35 or >35 years before interview/diagnosis, only other types of tobacco, non-smoker) and cigarette pack-years (log(py+1)) | | | | |
