# Supplementary material for: Lung cancer and socioeconomic status in a pooled analysis of case-control studies
Source: PLoS One. 2018 Feb 20;13(2):e0192999. doi: 10.1371/journal.pone.0192999 (PMC5819792; doi:10.1371/journal.pone.0192999)
Supplement: S1 Fig — Distribution of ISEI in male controls by study center. S1B Fig. Distribution of ISEI in female controls by study center. (DOCX) [file pone.0192999.s012.docx]

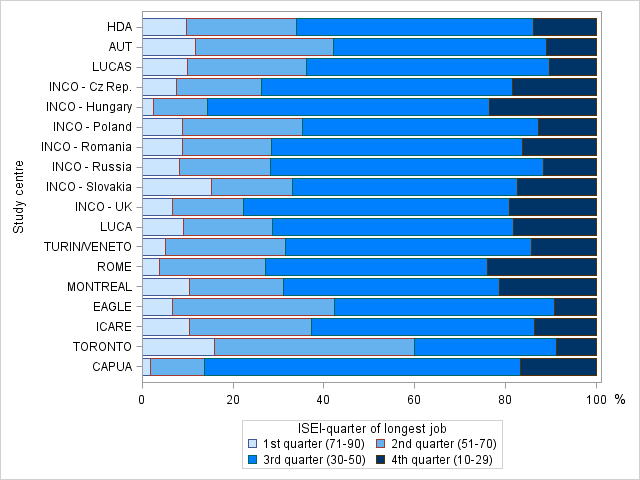


**S1A Fig.** Distribution of ISEI in male controls by study center.


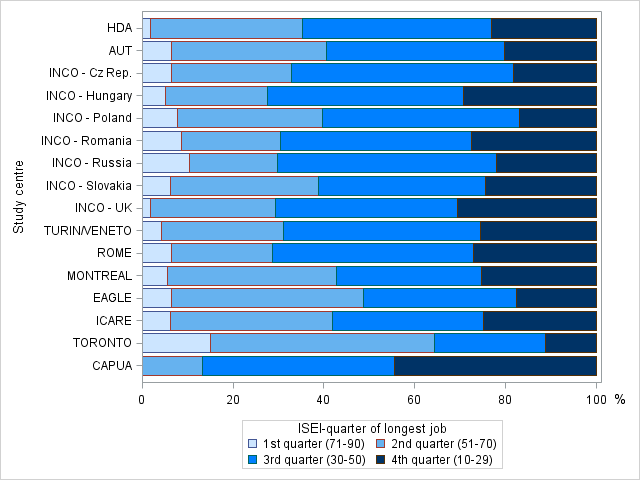


**S1B Fig.** Distribution of ISEI in female controls by study center.
