# Supplementary material for: Lung cancer and socioeconomic status in a pooled analysis of case-control studies
Source: PLoS One. 2018 Feb 20;13(2):e0192999. doi: 10.1371/journal.pone.0192999 (PMC5819792; doi:10.1371/journal.pone.0192999)
Supplement: S2 Fig — (DOCX) [file pone.0192999.s013.docx]

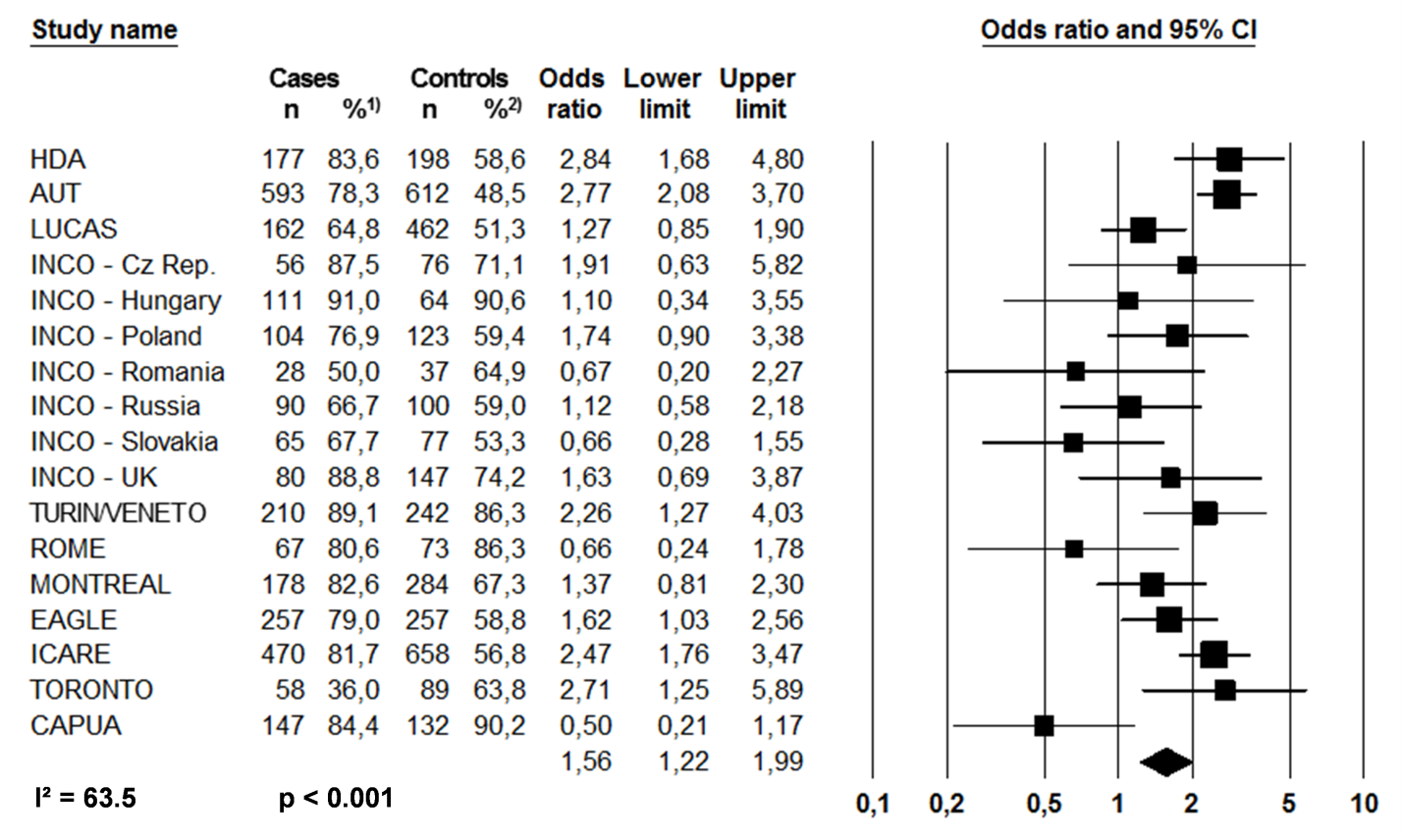


**S2 Fig.** Forest Plot of odds ratios by study center. ISEI (longest job, categories by quarters of ISEI-range): 4^th^ quarter (lowest) vs. 1^st^ quarter (highest).

Analysis restricted to men and without the LUCA study, adjusted for log(age), smoking status incl. time since quitting (current smoker, quitted 2-5, 6-10, 11-15, 16-25, 26-35 or >35 years before interview/diagnosis, only other types of tobacco, non-smoker) and cigarette pack-years (log(py+1))

^1)^ Proportion of exposed (lowest SES-category) cases

^2)^ Proportion of exposed (lowest SES-category) controls
